# Individual Assembly of Radical Molecules on Superconductors: Demonstrating Quantum Spin Behavior and Bistable Charge Rearrangement

**DOI:** 10.1021/acsnano.4c12387

**Published:** 2025-01-14

**Authors:** Chao Li, Vladislav Pokorný, Martin Žonda, Jung-Ching Liu, Ping Zhou, Outhmane Chahib, Thilo Glatzel, Robert Häner, Silvio Decurtins, Shi-Xia Liu, Rémy Pawlak, Ernst Meyer

**Affiliations:** † Department of Physics, University of Basel, Klingelbergstrasse 82, 4056 Basel, Switzerland; ‡ Institute of Physics (FZU), Czech Academy of Sciences, Na Slovance 2, 182 00 Prague 8, Czech Republic; § Department of Condensed Matter Physics, Faculty of Mathematics and Physics, Charles University, Ke Karlovu 5, 121 16 Prague 2, Czech Republic; ∥ Department of Chemistry, Biochemistry and Pharmaceutical Sciences, W. Inäbnit Laboratory for Molecular Quantum Materials, University of Bern, Freiestrasse 3, 3012 Bern, Switzerland

**Keywords:** Yu-Shiba-Rusinov states, superconductors, scanning tunneling microscopy, molecular manipulation, radical molecules, spin switch

## Abstract

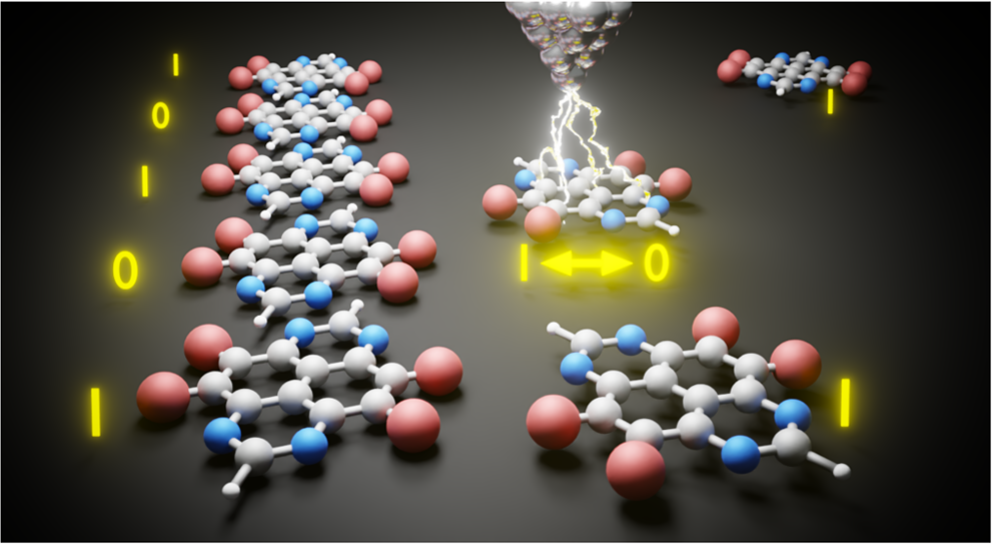

High-precision molecular manipulation techniques are used to control the distance between radical molecules on superconductors. Our results show that the molecules can host single electrons with a spin 1/2. By changing the distance between tip and sample, a quantum phase transition from the singlet to doublet ground state can be induced. Due to local screening and charge redistribution, we observe either charged or neutral molecules, which couple in a sophisticated way, showing quantum spin behavior that deviates from the classical spins. Dimers at different separations show multiple Yu-Shiba-Rusinov peaks in tunneling spectroscopy of varying intensity, which are in line with the superconducting two-impurity Anderson model, where singlet (*S* = 0) and doublet (*S* = 1/2) ground states are found. The assembly of chains of 3, 4, and 5 molecules shows alternating charge patterns, where the edge molecules always host a charge/spin. The tetramer is observed in two configurations, where the neutral site is moved by one position. We show that these two configurations can be switched by the action of the probing tip in a nondestructive manner, demonstrating that the tetramer is an information unit, based on single-electron charge reorganization.

## Introduction

Magnetic adatoms or molecules adsorbed on a superconductor have shown great potential for exploring electron spin interactions with the surrounding electron environment.^1^ These systems are promising for advancing applications in spintronics,^2^ quantum devices,^3^ and potentially enhancing the performance of superconducting diodes.^4^ Such impurities act destructively on Cooper pairs, breaking the time-reversal symmetry, leading to discrete states within the superconducting gap known as Yu-Shiba-Rusinov (YSR) states.^5^ These states can be studied using scanning tunneling microscopy (STM) and spectroscopy (STS), where they appear in tunneling spectra as pairs of resonances symmetrically positioned around the Fermi energy.^1^ YSR states were observed in various systems, including individual magnetic adatoms,^6−11^ transition metal complexes,^12−14^ rare-earth molecular magnets,^15^ paramagnetic molecules,^16,17^ metallic point contacts,^18,19^ and impurities embedded in unconventional superconductors.^20^

**Figure 1:**
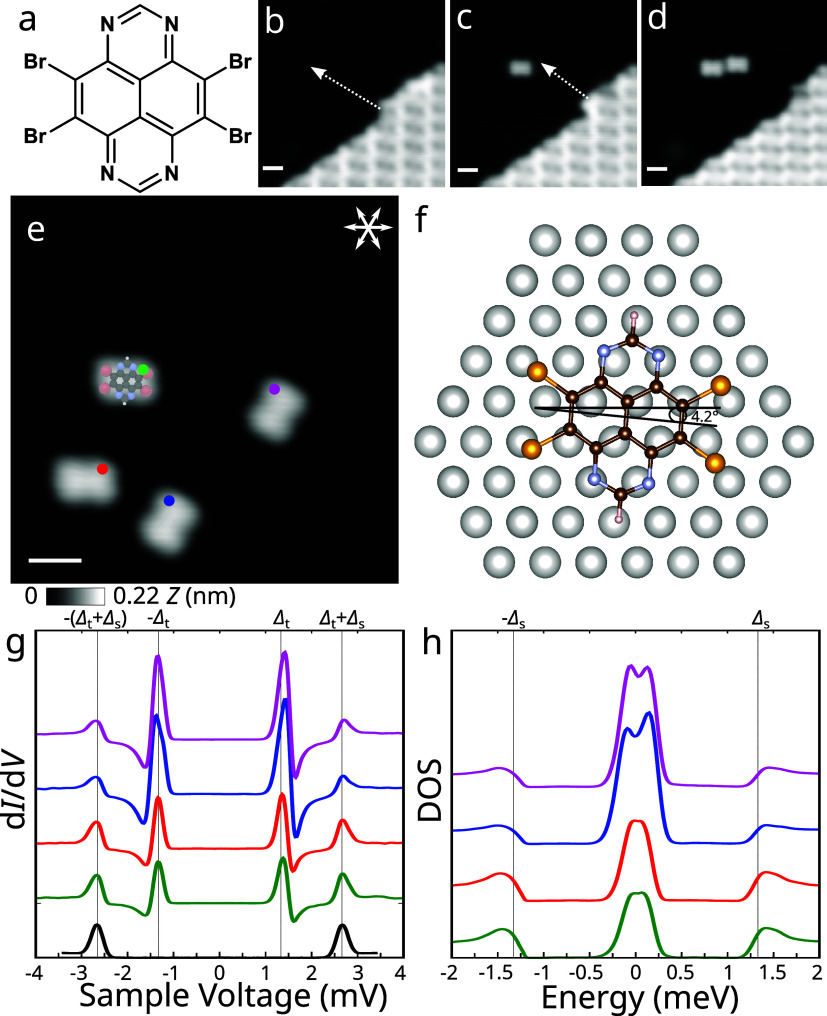
Tip manipulation and YSR spectroscopy of individual TBTAP radicals. (a) Chemical structure of the 4,5,9,10-tetrabromo-1,3,6,8-tetraazapyrene (TBTAP) molecule. (b–d) Creation of isolated TBTAP molecules by relocation from a molecular island through lateral manipulations (*I* = 100 pA, *V*_s_ = 500, 300, and 100 mV for b–d, respectively). (e) Four isolated TBTAP molecules exhibit preferred orientations aligned with densely packed directions of the surface with a small deviation of 4 ± 1° (*I* = 100 pA, *V*_s_ = 100 mV). (f) Equilibrium position of a TBTAP molecule on Pb(111) surface calculated using density functional theory (DFT). The molecule is oriented along the densely packed directions of the surface with a deviation of 4.2° between the ⟨110⟩ surface direction and one of the *c*_2_ axes of the molecule, in very good agreement with the experiment. (g) d*I*/d*V* spectra of isolated molecules measured at the positions marked by dots in panel e. In addition to the superconductor gap edge at Δ_t_ + Δ_s_ = 2.6 meV, clear additional resonances appear inside the gap at ±1.4 meV close to Δ_t_, indicating the presence of YSR states in isolated molecules. A spectrum for the pure Pb surface (black line) has been added for comparison. The spectra have been vertically shifted for clarity. (*V*_s_ = 5 mV; *I*_t_ = 0.4 nA, *A*_mod_ = 0.05 mV, *f* = 613 Hz). (h) Surface DOS of isolated molecules obtained by the deconvolution procedure from the tunneling spectra. YSR states lie very close to the Fermi energy, suggesting that the system is close to a QPT. The tip parameters for the deconvolution read Δ_t_ = 1.31 meV and γ_t_ = 0.04 meV. The scale bars in all figures represent 1 nm.

Most studies focusing on spinful molecules on superconducting surfaces use transition metal complexes in which the magnetic moment is mostly localized on the metal ion. Recent years have seen considerable efforts aimed at exploring organic magnets on metallic surfaces, driven by the increasing interest in manipulating the magnetic properties of organic free radicals.^21−24^ However, the study of magnetic radicals on superconducting surfaces remains a significant challenge and is still largely unexplored. Recently, a *D*_2*h*_-symmetric molecule 4,5,9,10-tetrabromo-1,3,6,8-tetraazapyrene (TBTAP), consisting of a tetraazapyrene backbone decorated by four bromine atoms (Figure 1a), has been reported to have a spin-1/2 magnetic ground state when adsorbed on either Ag(111) or Pb(111) surfaces.^25,26^ Manipulating the spin states of TBTAP molecules on the superconductor Pb could provide deeper insights into radical molecules on superconductors and position TBTAP as a compelling candidate for building molecular electronics.

Here, we present a low-temperature STM and STS study of TBTAP^•–^ molecules assembled on superconducting Pb(111), examining systems from single molecules to five-unit chains. Starting with single-molecule analysis, we accurately modeled dimers, leading to insights on how information can be encoded in tetramer chains by switching their charge states. This approach revealed that TBTAP^•–^ molecules are near a quantum phase transition (QPT) on the Pb(111) surface, where the QPT between singlet and doublet states can be controlled by adjusting the STM tip distance. Our experimental results aligned with theoretical predictions based on DFT and the superconducting impurity Anderson model. The magnetic states of TBTAP pairs were further manipulated by altering their distance and orientation, transitioning from individual molecules to correlated dimers, tuning YSR states, and inducing a QPT with strong ferromagnetic correlations. Finally, the TBTAP chains of three to five units were created, showing that information can be encoded by switching charge states, with frustrated dimer structures at the ends of even-length chains playing a key role.

## Results and Discussion

### Manipulation and Tunneling Spectroscopy of Individual TBTAP Radicals

TBTAP molecules have recently been reported to exhibit a radical TBTAP^•–^ state within self-assembled monolayers on Pb(111).^26^ The molecules within the 2D assembly are aligned in registry with the Pb(111) surface and the structure is stabilized by a combination of halogen bonds and C–N···Br–C bonds. Both type-I of type-II geometries occur.^27−29^ The radical has a ground state *S* = 1/2 that gives rise to YSR states within the superconducting gap. To study the intrinsic behavior of individual TBTAP molecules, they were removed by the STM tip from a molecular island (Figure 1b–d). Each molecule is adsorbed along the close-packed surface direction ⟨110⟩, with a small deviation of 4 ± 1°. This behavior is in good agreement with the DFT result (Figure 1f), which predicts the molecule to lie ≈330 pm above the surface at the angle of 4.2°. The magnetic state was verified by measurements using a gradually increasing out-of-plane magnetic field up to 1 T at *T*_exp_ = 2.2 K. The magnetic field used is strong enough to suppress the superconductivity in the tip and the substrate. The STS results show a well-developed Kondo peak. Its Frota fit^30^ gives an estimated Kondo temperature *T*_K_^FF^ = 7.5 K (see Supporting Note 4).

Differential conductance (d*I*/d*V*) spectra were recorded at *T*_exp_ = 1 K on four molecules at positions marked by dots in Figure 1e. Due to the use of a superconducting Pb tip, the tunneling spectra are convolutions of the density of states (DOS) of the tip (t) and of the substrate (s) and all energies are shifted by the tip gap Δ_t_. The spectra are plotted in Figure 1g, together with a spectrum of a bare Pb surface. In addition to the coherence peaks at Δ_t_ + Δ_s_ = ±2.6 meV, a single pair of slightly asymmetric peaks emerge at *V* = ±1.4 mV. The asymmetry of the peaks is due to the Coulomb potential which breaks the particle-hole symmetry^5^ and is a hallmark of the single-electron nature of the involved tunneling process.^31^ The character of the YSR states is better illustrated on the surface DOS obtained from the tunneling spectra by deconvolution (see Supporting Note 2) plotted in Figure 1h, which shows the YSR states that lie close to the Fermi energy. This behavior indicates that the system is very close to an impurity QPT between the doublet and singlet ground states.^32^ The crossing of the YSR states at the Fermi energy is a hallmark of the QPT transition, as the YSR energies are the differences between the energy of the ground state and the first excited state, which cross and exchange order at QPT.^5,33^

**Figure 2:**
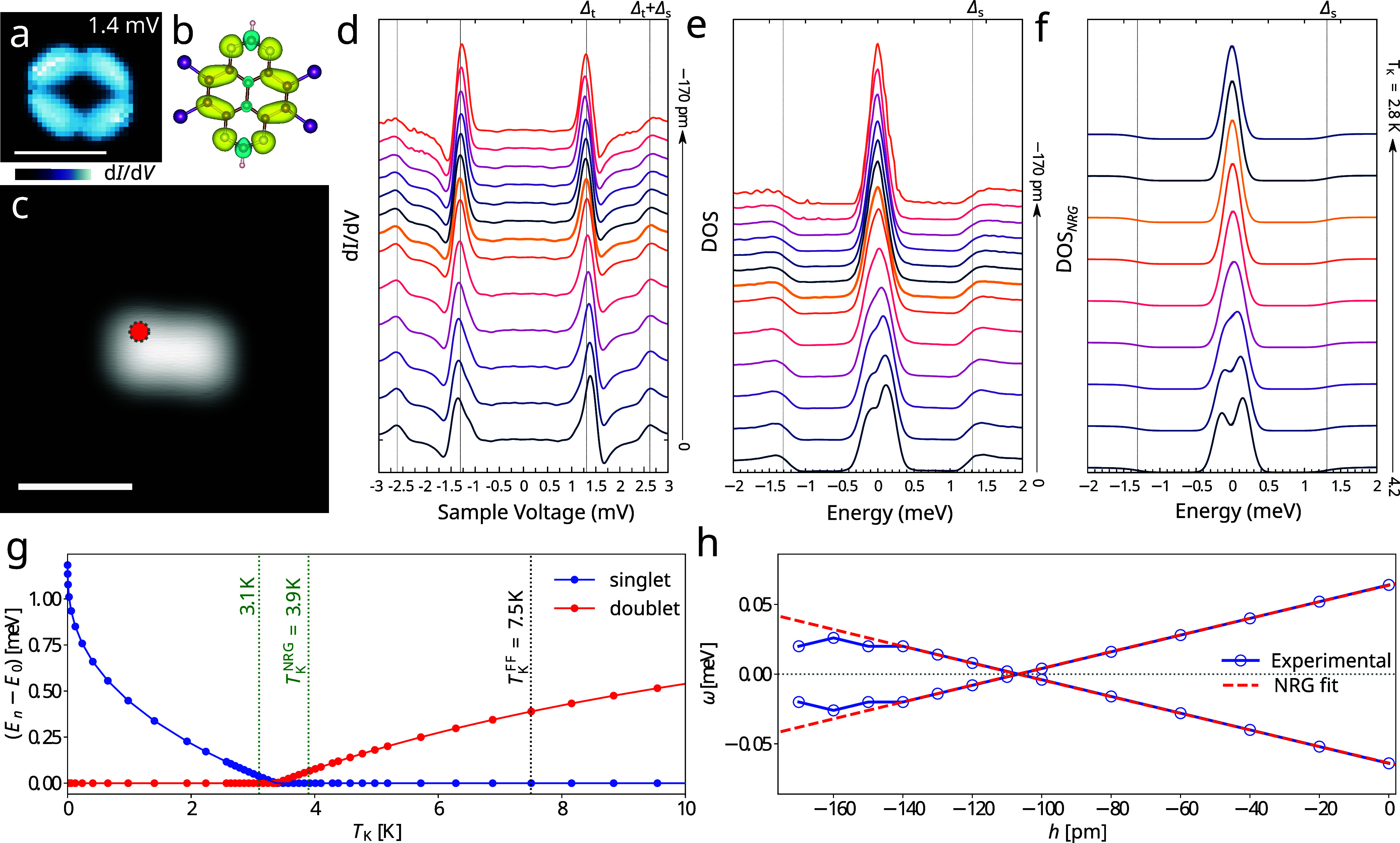
Quantum phase transition of an individual TBTAP radical. (a) Grid map of d*I*/d*V* at *V* = 1.4 mV, close to the YSR resonance. (b) Spin density map of the charged TBTAP molecule on the Pb substrate calculated using DFT. The yellow (turquoise) color marks the positive (negative) spin density. The orientation of the molecule matches panel a. (c) A high resolution STM image (*V*_s_ = 0.1 V and *I*_t_ = 0.1 nA) of an individual TBTAP molecule. (d) Differential conductance spectra at different tip heights *h* measured using the superconducting Pb tip. The initial tip position was set at *I* = 100 pA, *V*_s_ = 5 mV above the molecule, followed by a gradual approach toward the molecule. The Fermi energy value was corrected in each data set by a small shift determined from the positions of the coherence peaks. (*V*_s_ = 6 mV, *A*_mod_ = 0.02 mV, *f* = 613 Hz). (e) Surface DOS obtained using the deconvolution procedure from d*I*/d*V* data in panel d. The thicker yellow lines in panels d and e represent data for *h* = −110 pm, closest to the QPT. The tip parameters for the deconvolution read Δ_t_ = 1.31 meV and γ_t_ = 0.04 meV. (f) Surface DOS calculated using NRG for different values of the Kondo temperature *T*_K_ for *U* = 200 meV and slightly away from half-filling (ε = −20 meV) to simulate the asymmetry in the differential conductance spectra. (g) Subgap states, respectively the differences between the first excited state and the ground state energies, of the SC-SIAM as functions of *T*_K_ calculated using NRG. The blue (red) points represent the singlet (doublet) state. The black dashed line marks the *T*_K_ obtained from the fit of the normal-state Kondo peak with the Frota function. The green dashed lines mark the range of *T*_K_ corresponding to the change of the vertical distance of the scanning tip from the molecule, fitted from the position of the YSR peaks in d*I*/d*V* via SC-SIAM solved by NRG. (h) Positions of the subgap state maxima extracted from the experimental d*I*/d*V* data and shifted by Δ_t_ (blue symbols). The red dashed line represents the fit by NRG as described in the text. The energies were averaged over the positive and negative values to increase the quality for small distances.

Figure 2a displays a local density map of a molecule at the YSR position of 1.4 mV (see Supporting Note 1). This map unveils the spatial distribution of the YSR state, which has the same symmetry as the spin density of a TBTAP^•–^ calculated using DFT (Figure 2b). The YSR states are delocalized over the backbone of aromatic rings. This is in contrast to experiments performed on organometallic complexes, where the YSR states are localized on the metallic ion. However, no coherent long-range extent of the YSR state away from the molecule was observed. This can be attributed to the three-dimensional nature of the Pb(111) substrate, in which the spatial extent of the YSR states is strongly suppressed compared to experiments conducted on quasi-two-dimensional substrates such as NbSe_2_.^34^

### Quantum Phase Transition of an Individual TBTAP Molecule

The YSR states are close to the Fermi energy, which implies that the system is naturally set in the vicinity of a QPT. This transition can be achieved by different means, including using different adsorption sites^35^ or applying mechanical forces with an STM tip.^36^ Achieving QPT in molecules could provide new approaches for improved control and tunability in molecular spintronics.^37−40^ To demonstrate the transition, a series of STS measurements was performed on the molecule while varying the tip height at the position marked by a red dot in Figure 2c. The controllable motion of molecules during lateral manipulation suggests the existence of an attractive force between the molecule and the tip. This force lifts the molecule from the substrate and has the potential to fine-tune the coupling strength between the molecule and its environment. The scanning tip was approached within 170 pm of its initial position (*I* = 100 pA, *V* = 10 mV). The resulting spectra are plotted in Figure 2d. The YSR position decreases until *h* ≈ −110 pm and then increases again, suggesting the crossing of the YSR states. To better visualize this crossing, a deconvolution was performed to obtain the surface DOS (Figure 2e). The position of the YSR state changes from 60 to −30 μeV, indicating a QPT from a singlet ground state at large distances to a doublet ground state at small distances.

**Figure 3:**
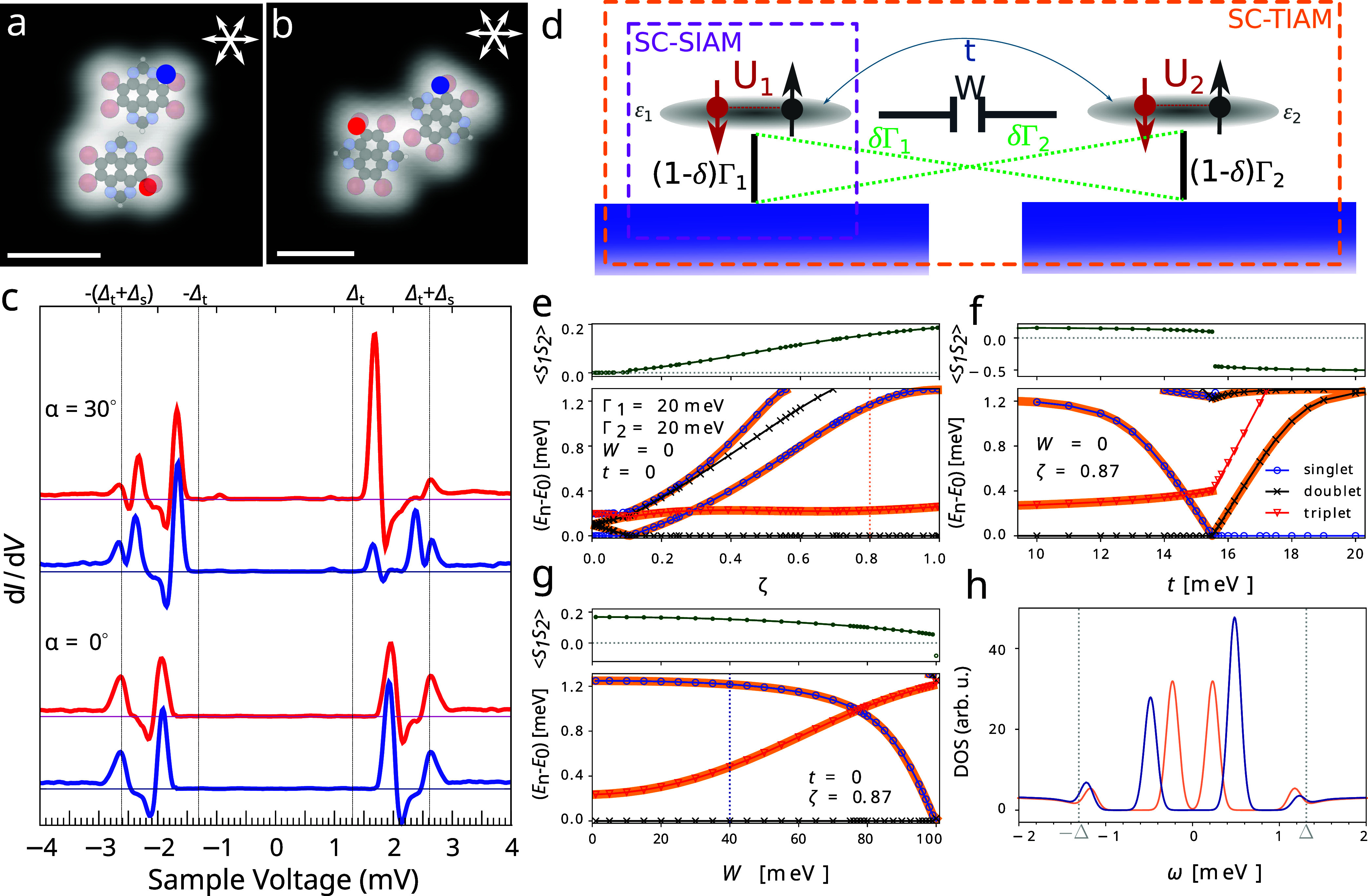
Theoretical analysis of the YSR states in TBTAP dimers. (a, b) Two configurations of molecular dimers constructed by lateral manipulations (*V*_s_ = 0.1 V and *I*_t_ = 60 pA for a; *V*_s_ = 50 mV and *I*_t_ = 0.1 nA for b). (c) Tunneling spectra of molecules from panels a (α = 0°) and b (α = 30°) measured at positions marked by the red and blue dots. The spectra have been vertically shifted for clarity. (d) Illustration of SC-SIAM (violet frame) for single molecule and SC-TIAM for the dimer (orange frame). For SC-SIAM we set δ = 0 and Γ_1_ ≡ Γ is the total coupling of the impurity to both the substrate and the scanning tip (not shown). The SC-TIAM scheme shows all relevant couplings analyzed in the main text. For δ = 1/2 the model describes a local two-level impurity that corresponds to small intermolecular distance. Here, the direct hopping between the impurity orbitals *t* and the interimpurity capacitive coupling *W* can be significant. In the opposite limit δ = 0 describes two distant molecules, each effectively coupled to its own substrate, for which *t* → 0 and *W* → 0. (e–g) Bottom panels: YSR states of a dimer described by SC-TIAM calculated using NRG as functions of the intermolecular coupling parameter  (panel e), direct hopping *t* (panel f) and capacitive coupling *W* (panel g), respectively. The blue circles, black crosses, and red triangles denote the singlet, doublet, and triplet states, respectively. Orange lines denote the actual YSR states which appear in the STS data, that is, transitions that are not forbidden by the Δ*s*_*z*_ = ±1/2 parity selection rule. Top panels: The respective interimpurity spin–spin correlation function ⟨***S***_1_***S***_2_⟩ in units of ℏ^2^. Positive values signal a ferromagnetic effective RKKY exchange, and negative values mark an antiferromagnetic order, where electrons form an entangled singlet state. (h) Surface DOS calculated using NRG for the same parameters as in panel e for ζ = 0.8 and *W* = 0 (salmon) and panel g ζ = 0.87 and *W* = 40 meV (dark blue). This value of the interimpurity coupling shifts the YSR energies close to the experimental values in the upper part of panel c (α = 30°).

This behavior can be described using the superconducting single impurity Anderson model (SC-SIAM),^41^ which is known to provide reliable insights into the physics of superconducting impurity systems.^42^ The system can be described as a single quantum level tunnel-coupled to two superconducting leads, the surface and the tip. An illustration of SC-SIAM is shown in Figure 3d and more details are provided in the Supporting Note 3. From now on, we assume that Δ_t_ = Δ_s_ = Δ. The decrease in tip height increases the coupling to the tip Γ_t_, but decreases the coupling to the substrate Γ_s_ by lifting the molecule. As Γ_s_ ≫ Γ_t_ this leads to a decrease in total coupling Γ = Γ_s_ + Γ_t_. Note that at small distances the force between the tip and the molecule eventually changes to repulsive, again leading to an increase in Γ_s_.^36^

The governing scale of the singlet-doublet QPT in SC-SIAM for the parameter regime of the experiment is the Kondo temperature *T*_K_.^41,43^ The ground state changes from a singlet to a doublet at the critical value *T*_K_^c^ that can be approximated by^43^*k*_B_*T*_K_^c^ ≈ Δ/(e^5/3^–1) ≐ 0.23Δ. For Δ = 1.31 meV this gives *T*_K_^c^ = 3.5 K. The small YSR energies suggest that the system is close to the QPT already at the initial position of the tip. Therefore, it is expected that *T*_K_ is close to *T*_K_^c^ in the whole measured range of the tip height. To test this conjecture, the analysis of the experimental data using numerical renormalization group^44^ (NRG) solution of SC-SIAM (see the Methods section) was performed, which provides an alternative way to extract the *T*_K_ of the setup. Figure 2g shows the energy of the subgap states with respect to the ground state energy *E*_0_ as functions of *T*_K_ for a half-filled impurity. The differences ω^±^ = ± |*E*_1_ – *E*_0_| are the YSR energies, which appear as subgap peaks in the surface DOS. YSR states cross at the Fermi energy at *T*_K_^c^ ≃ 3.5 K as expected from the above estimate.

The YSR energy extracted from the tunneling spectra as the maximum of the in-gap peak as a function of the tip distance *h* is shown in Figure 2h. Note that *h* = 0 is just a reference value that refers to 650 ± 50 pm above the molecule. The YSR energy depends almost linearly on *h* up to very small distances, where the YSR states turn again toward the Fermi energy. This behavior is a result of the force between the tip and the molecule changing from attractive to repulsive, pushing the system back toward the QPT. The values in the linear regime around the QPT can be utilized to obtain a better estimate of *T*_K_ by an NRG fit. However, this requires a mapping between *T*_K_ and *h*. To that end, Wilson’s formula for the Kondo temperature, eq 1 was utilized, together with a linearization procedure explained in the Supporting Note 5. In the vicinity of QPT this leads to a linear relation *h* = (Γ – Γ_0_)/α̃, where Γ_0_ and α̃ are parameters that can be fitted from the experimental data. The NRG results, marked by the dashed red lines in Figure 2h, overlap with the experiment and can be used to extract Γ_exp_ and consequently *T*_K_. Assuming *U* = 200 meV,^25,26^ this leads to values of Γ_exp_ between 18.2 and 19.8 meV in the linear regime that gives *T*_K_^NRG^ between 3.1 and 3.9 K.

Figure 2f shows the surface DOS calculated using NRG for values of *T*_K_ from the extracted interval. The evolution of DOS is in agreement with the experimental results. The difference in weights of the bands above the gap between theory and experiment can be attributed to an experimental background not included in the SC-SIAM analysis.

Estimation of *T*_K_ for the single-molecule system revealed an inconsistency between the Frota fit and the NRG result. There are multiple mechanisms that can lead to this disagreement, e.g., NRG relies on estimates of model parameters such as Coulomb repulsion. However, due to Kondo universality, the dependence of YSR state on *T*_K_ from Figure 2g and consequently also the NRG fit are very robust when varying the model parameters within experimentally meaningful limits. On the other hand, it has already been shown^45^ that a simple Frota fit of the Kondo peak significantly overestimates the Kondo temperature when *T*_K_ is comparable to the experimental temperature, which is our case as *T*_exp_ = 2.2 K for the normal state measurement. Thus, it can be argued that the NRG fit method introduced here provides a more accurate estimate of the actual Kondo temperature compared to the Frota fit, suggesting that the system is inherently very close to a QPT.

### Theoretical Analysis of the YSR States in TBTAP Dimers

Another way to manipulate YSR states in molecular systems is by engineering setups consisting of two impurities. Two different types of molecular dimers were constructed using lateral manipulation technique. A density map of a dimer consisting of molecules with the same orientation is shown in Figure 3a. This configuration is stabilized by noncovalent interactions such as N···H hydrogen bonds. Tunneling spectra, recorded at positions marked by the dots in Figure 3a, are plotted at the bottom of Figure 3c (α = 0°) by respective colors. Both molecules exhibit almost identical spectra that show a single pair of YSR states at *V* = ±1.9 mV. The evolution of the position of the peaks between the limit of two isolated molecules and the dimer is briefly discussed in the Supporting Note 8.

A different configuration of molecules is shown in Figure 3b. In this configuration, stabilized by Br···Br and Br···N halogen bonds, the *c*_2_ axes of the two molecules form a 30° angle. It should be noted that the orientation of one molecule in this dimer is rotated by 30° compared to the isolated molecule, due to the change in adsorption orientation caused by the adjacent molecule. The corresponding tunneling spectra, plotted in the upper part of Figure 3c (α = 30°), exhibit two pairs of YSR states at *V*_1_ = ±1.7 mV and *V*_2_ = ±2.5 mV. The deconvolved surface DOS for both configurations can be found in the Supporting Note 8.

The behavior of the dimer system was analyzed using the superconducting two-impurity Anderson model (SC-TIAM). Its schematic representation is shown in Figure 3d and a detailed description can be found in Supporting Note 6. In general, the effect of the distance between impurities should be encoded in SC-TIAM via complex hybridization terms.^46^ However, for a constant substrate DOS this effect can be parametrized by tunneling cross-terms (green lines in Figure 3d). Here, δ ∈ ⟨0,1/2⟩, respectively  (see Supporting Note 6), is a parameter that indirectly encodes the intermolecular distance. The value δ = 0 corresponds to a system of two distant impurities, each effectively coupled to its own substrate. The other limit δ = 1/2 represents a single two-level impurity coupled to a single substrate.^47,48^ By varying δ, the model can tune the intermolecular correlations through the substrate, potentially leading to both effective coupling and exchange interactions of the RKKY type.^46,49^

Following the analysis of the TBTAP molecule, the coupling was fixed to Γ_1_ = Γ_2_ = 20 meV, setting each of the two distant molecules to a singlet ground state. An alternative analysis, in which different ground states are assumed for the molecules as a result of the presence of a scanning tip, leads to equivalent results, as presented in the Supporting Note 7. Bottom panel of Figure 3e shows the in-gap many-body energies of SC-TIAM with respect to the ground-state energy as functions of ζ. The direct hopping *t* and the capacitive coupling *W* was set to zero, that is, the molecules are correlated only through the substrate. The top panel shows the corresponding spin–spin correlation function ⟨***S***_1_***S***_2_⟩ for the ground state.

For nearly decoupled impurities (ζ → 0) a combined ground state is a singlet^50^ and the spins are very weakly correlated. There is only a single YSR state at very low energies, as individual molecules are close to a QPT. With increasing ζ (decreasing distance), the YSR state splits into two that lie very close to each other; therefore, they might not be distinguishable in the STS data. At ζ ≃ 0.1, the system undergoes a QPT and the ground state changes to a doublet. A short discussion on the differences in the spin structure of the dimer in the singlet and doublet ground states can be found in Supporting Note 6. Doublet ground state allows for richer in-gap spectrum: Up to three pairs of YSR states are predicted for intermediate values of ζ and two pairs for large ζ (small distance). The results suggest that the ground state for both dimers is a doublet and the inner pair of YSR states in the experiment is due to the doublet-triplet transition emerging as a result of the correlations between the molecules through the surface. The YSR energies are shifted to larger values with decreasing distance, in agreement with the experimental result. At ζ → 1, the outer pair of YSR states moves toward the edge of the gap and eventually vanishes from the spectrum.

This analysis shows that SC-TIAM qualitatively reproduces the experimental results. However, the YSR state energies from NRG do not quantitatively match the experimental data. These discrepancies can be adjusted by assuming finite *t* and *W*. Both the intermolecular coupling *W* and the direct hopping *t* are expected to be significant only at small distances. Figure 3f,g show the effect of both for ζ = 0.87. A hopping with energy smaller than or comparable to Γ can fine-tune the YSR energies where the singlet state quickly approaches and finally crosses first the triplet state and then the doublet as *t* increases. Consequently, a relatively small *t* can switch the dimer from a doublet to a molecular singlet ground state. Conversely, as *W* increases, the difference between the triplet and doublet state energies widens, as illustrated in Figure 3g. This significantly shifts the position of the inner YSR peaks to higher energies, which is in compliance with experimental results. The higher weight of the inner pair of YSR states compared to the outer pair shown in the surface DOS plotted in Figure 3h is consistent with the experimental observation. A similar approach to obtain spectra in compliance with the configuration in Figure 3a is discussed in the Supporting Note 8.

Note that both *t* and *W* can independently trigger a QPT from molecular doublet to the singlet ground state. At the critical point, ⟨***S***_1_***S***_2_⟩ changes discontinuously from positive to negative values, signaling a transition from effective ferromagnetic to effective antiferromagnetic exchange coupling. However, one must be careful with the interpretation of ⟨***S***_1_***S***_2_⟩ in the doublet phase. Here, the spin of the molecules is partially screened by the conducting electrons. In the idealized case, one of the spins is fully screened and the other stays unscreened, leading to a vanishing correlator. A finite value can still emerge as a result of charge fluctuations. Due to the partial screening of the spin, the correlator is strictly smaller than 1/4, i.e., the asymptotic value of strong ferromagnetic exchange expected in the triplet state. In this respect, the observed value ⟨***S***_1_***S***_2_⟩ ≈ 0.2 is large, pointing to effective ferromagnetic exchange with partially screened spins, as also discussed in Supporting Note 6.

The spectrum of the dimer also depends on the relative orientation of the molecules. Despite a similar distance, the YSR state splits only in some cases. The effect of magnetic anisotropy can be ruled out, since the isolated TBTAP^•–^ molecules are in spin-1/2 ground state and are free of any heavier atoms. Similar splitting of the YSR state in molecular dimers was also observed in Co phthalocyanine dimers^12^ and was attributed to ferromagnetic coupling via a phenomenological model. This conclusion is consistent with small positive values of ⟨***S***_1_***S***_2_⟩ in our SC-TIAM analysis. However, in order to understand the influence of the orientation of the molecules on the splitting, a detailed ab initio analysis would be required. Admittedly, the formula that would connect ζ to the distance between the molecules and their relative orientation would be complicated. For example, the theory of two classical magnetic impurities in a superconductor predicts a complex dependence of the effective hybridization between the impurities on the distance.^51^ Similar effects of distance can be expected in SC-TIAM with energy-dependent Γ.

### Switching the Charge State of Short Molecular Chains

**Figure 4:**
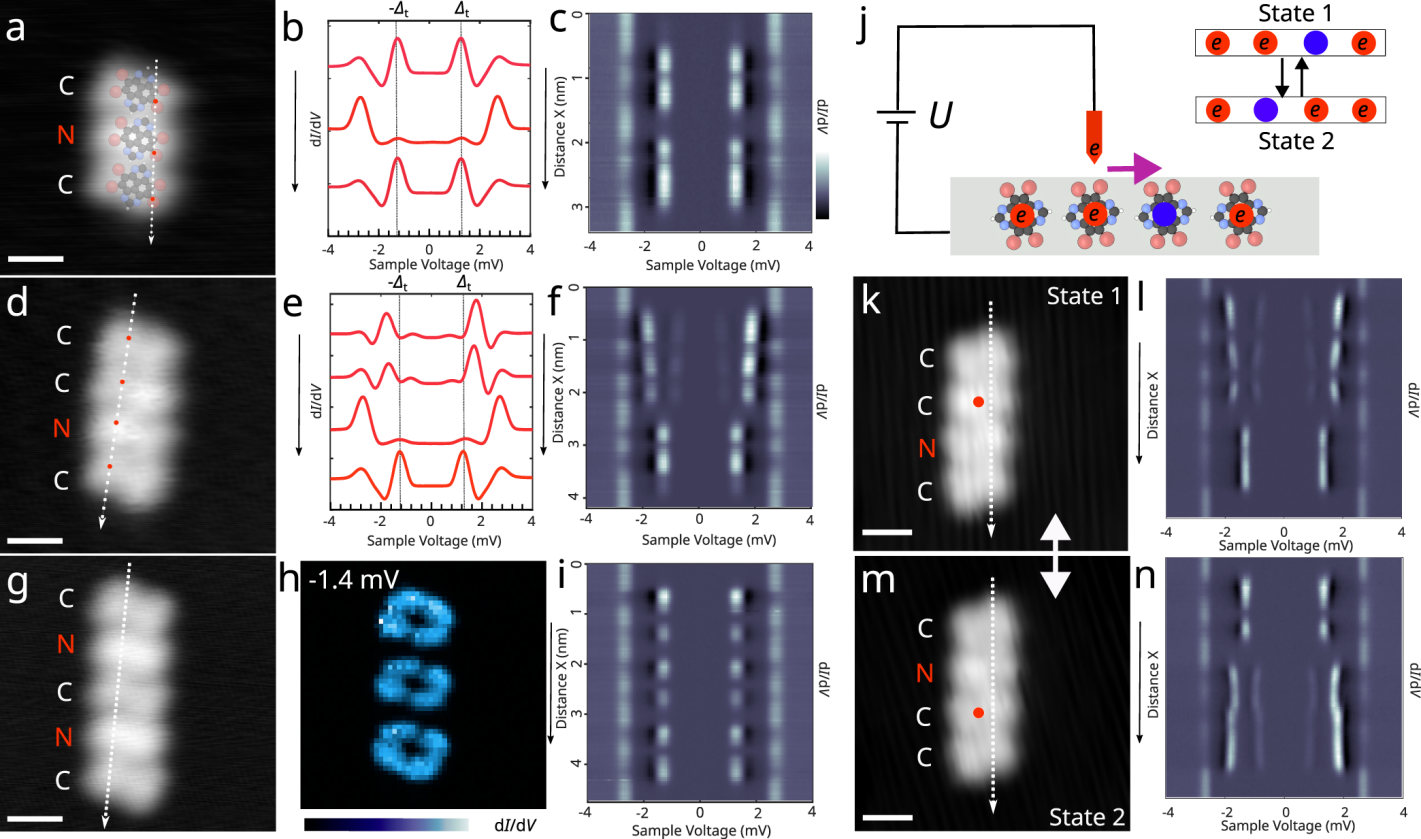
Controlling molecule’s charge states in constructed chains. (a) Formation of a trimer chain via lateral manipulation, coupled by N···H hydrogen bonding between molecules. The C (N) label denotes a charged (neutral) molecule. (b) Differential conductance spectra at the positions indicated by red dots in panel a, showing distinct electronic characteristics: a single YSR peak pair at *V*_s_ = ± 1.4 mV for the end molecules and absence of YSR peaks in the central molecule. (c) d*I*/d*V* cross-sectional analysis along the trimer, indicated by a white arrow in panel a. The high conductance at *V* = ±2.6 mV denotes the coherence peaks of Pb(111), while the high conductance at *V* = ± 1.4 mV marks the YSR states. (d) A tetramer chain assembled through lateral manipulations. (e) d*I*/d*V* spectra for the tetramer chain show a pair of YSR peaks at *V*_s_ = ±1.9 mV for the top two molecules, the absence of YSR states in the third, and a YSR peak pair at *V*_s_ = ±1.4 mV for the end molecule. (f) d*I*/d*V* cross-sectional analysis along the tetramer chain. (g) A pentamer chain crafted by lateral manipulation. (h) Grid maps of the d*I*/d*V* measurements at *V*_s_ = −1.4 mV highlight localized electronic properties. (i) d*I*/d*V* cross-sectional view across the pentamer, measured along the white arrow in panel g. (*V*_s_ = 0.1 V; *I*_t_ = 0.1 nA for a, d and g; spectra parameters: *V*_s_ = 5 mV, *I*_t_ = 0.1 nA, *A*_mod_ = 0.03 mV and *f* = 613 Hz). (j) Schematic representation of four TBTAP molecules on a Pb(111) surface. A negatively charged tip is positioned above one of the negatively charged TBTAP molecules. The repulsive force induces a charge transfer to the adjacent molecule, as illustrated by the arrow. The inset illustrates the equivalent states of a tetramer chain. (k, m) Another tetramer chain crafted to test the possibility of switching the assembly between the two possible equivalent states. When the tip is positioned at the location marked by the red circle and moved 100 pm closer to the molecule, the electron is transferred to the adjacent uncharged molecule (*V*_s_ = 80 mV; *I*_t_ = 0.1 nA). (l) d*I*/d*V* cross-sectional view along the tetramer indicated by a white arrow in panel k, showing the position of the dimer on the top two molecules. (n) d*I*/d*V* cross-sectional view along the tetramer indicated by a white arrow in panel m shows the dimer localized on the bottom two molecules. Data were acquired at a sample temperature of 2.2 K.

Further control over the YSR states can be achieved in longer chains of molecules. Figure 4a shows a molecular chain consisting of three molecules connected by hydrogen bonds, all oriented in the same direction. Tunneling spectra and a d*I*/d*V* cross-section are plotted in Figure 4b,c. They show that the first and third molecules exhibit a YSR state at *V* = ± 1.4 mV, similarly to the case of an isolated molecule. However, the spectrum measured over the central molecule is free of any in-gap features, except for faint peaks that can be attributed to the extension of the YSR states of adjacent molecules. This points to the loss of the radical nature of this molecule (Supporting Note 9).

The tetramer chain was constructed by adding another molecule with the same orientation (Figure 4d). The tunneling spectra (Figure 4e) and the d*I*/d*V* cross-section (Figure 4f) reveal that the top two molecules exhibit YSR states at *V* = ± 1.9 mV, consistent with those seen in the dimer (Figure 3a,c). The third molecule is in a neutral state, whereas the fourth molecule behaves as isolated with YSR states near the Fermi energy. The YSR energy shows a slight difference of *V* ≈ 0.1 mV between the first and second molecules, likely due to a weak coupling between the second and fourth molecules. Note that similarly to the case of a trimer, the molecules at the end of the chain always retain their radical nature.

Inspired by the different behavior observed in the trimer and tetramer chains, a pentamer was constructed by adding yet another molecule to the tetramer (Figure 4g). The d*I*/d*V* cross-section (Figure 4i) shows that only the first, third, and fifth molecule hosts a YSR state at eV ≈ Δ. A grid map close to the YSR position of −1.4 mV was measured (Figure 4h), confirms that the spatial shape of the YSR states in the odd-numbered molecules is the same as in individual molecules (Figure 2a).

These results indicate that the radical character of a specific TBTAP molecule can be controlled through the interaction with other molecules. The presence of two adjacent charged molecules is required for the change of charge state, since the first and last molecules are always in the radical TBTAP^•–^ state. These molecules also behave as isolated with YSR states close to the Fermi energy, despite being in contact with a neutral TBTAP^0^ molecule. This indicates that the shift of the YSR states in dimers is the result of the magnetic interaction of the spins on both molecules, as assumed by the analysis of the dimer using SC-TIAM. The loss of the charge can be explained by the presence of an additional electrostatic field induced by the two adjacent charged molecules, which shifts the SOMO energy of the molecule above the chemical potential of the substrate, leaving the molecule in the neutral state. A similar effect was also observed in TBTAP monolayers on Pb(111)^26^ and in the TCNQ monolayers on Au(111).^52^ As a result of this behavior, chains of TBTAP molecules create periodic structures of charged and neutral units. Such an order can be achieved only in chains of odd lengths, as the first and last molecules are always charged. In chains of even length, this is compensated by emergence of a dimer structure on one of the ends. Such a system is in a frustrated state with two equivalent charge configurations, as the dimer can form on either end of the chain. External perturbations may induce charge transfer to an adjacent molecule in the chain and switch the system to the other energy minimum, as depicted in Figure 4j.

To test this concept, another tetramer chain was constructed (Figure 4k,m). The d*I*/d*V* cross-sectional view along the chain (Figure 4l), indicated by a white arrow in panel k, displays the dimer structure appearing on the top two molecules. When the tip is positioned at the location marked by the red circle and moved 100 pm closer to the molecule, the chain is switched to the other energy minimum and the d*I*/d*V* cross-sectional view (Figure 4n) now shows the dimer localized on the bottom two molecules, implying successful charge transfer. This switching process has been repeatedly performed (not shown), demonstrating its reversibility and the stability of the charge configuration. This suggests a possibility of encoding information in such assemblies.

## Conclusions

We demonstrated that metal-free TBTAP molecules on Pb(111) are easy to manipulate using the STM tip, they always adsorb to the surface in the same manner, and can be combined to create stable assemblies. Individual molecules are in a radical state with spin 1/2, which gives rise to well-developed YSR states. These states are spatially delocalized along the organic backbone of the molecule. Although other metal-free organic radicals exist,^21,22,53^ they do not allow for a higher level of manipulation. The individual TBTAP molecules are close to a singlet-doublet impurity QPT that can be induced by an STM tip. The properties of this molecule can also be tuned by the presence of the second TBTAP molecule: the intermolecular distance tunes the YSR energy, while the splitting of the YSR states into two pairs can be engineered by changing their relative orientation. Yet another type of control can be achieved in longer chains of molecules. Chains with odd and even number of constituents behave differently. Odd-numbered chains exhibit a periodic structure of YSR states localized on every second molecule, always including the ends of the chain. Chains of even length display a more complicated behavior in which a molecular dimer is formed on one of the ends. The dimer can be transferred from one end to the other by external electric field induced by the presence of the scanning tip, opening the possibility to store information in these structures. Together, the different assemblies of TBTAP molecules can be utilized as highly tunable building blocks for superconducting molecular quantum technologies.

## Methods

### Sample Preparation

The Pb(111) surface (MaTeck GmbH, 99.999%) was cleaned by cycles of Ar ion sputtering and annealing. TBTAP molecules were prepared according to the literature procedure^54^ and sublimated at ≈440 K with the substrate kept at ≈100–150 K.

### Lateral Manipulation

To manipulate isolated TBTAP molecules on the Pb(111) surface, we employed a lateral manipulation process commonly used to move molecules on metal surfaces.^17,55,56^ Initially, the STM tip was positioned directly above the designated molecule using normal scanning parameters (*I* = 100 pA, *V*_s_ = 100 mV). Subsequently, the STM tip was lowered to approximate position of the target molecule employing *I* = 3 nA and *V*_s_ = 3 mV, while disabling the feedback mechanism. The tip was then moved to the desired location at a speed of 200 pm/s, while simultaneously pulling the molecule along with it. Finally, upon reaching the target location, the feedback was reactivated and the scanning parameters reverted to *I* = 100 pA, *V*_s_ = 100 mV.

### STM/STS Experiments

STM and STS experiments were performed with a low-temperature (1 K) Joule–Thomson STM/AFM microscope (purchased from Omicron GmbH) in ultrahigh vacuum (UHV) of ≈10^–10^ mbar operated with Nanonis RC5e electronics. Differential conductance, d*I*/d*V*, spectra were recorded with an internal Nanonis lock-in amplifier using modulation amplitudes indicated in the figure captions. The measurements were performed with a pure Pb tip to enhance the energy resolution beyond the thermal limit.

### Ab Initio Calculations

The density functional theory was used to find the equilibrium position of a TBTAP molecule adsorbed onto Pb(111) surface. Calculations were carried out using Turbomole 7.5.1^57^ridft code utilizing the def2-TZVP (triple-ζ) basis set and B3-LYP exchange-correlation functional with DFT-D3 (Becke–Johnson) dispersion correction. The Pb surface was modeled as a slab of three atomic layers, 60 Pb atoms in total. The system was charged by one electron to simulate charge transfer to the molecule.

### Deconvolution of the Tunneling Spectra

A numerical deconvolution procedure was used to obtain the surface density of states from the d*I*/d*V* data. We utilized the maximum entropy method^58^ implemented in a modified ana_cont package^59^ using a flat default model of the same width as the input data. The optimal value of the hyperparameter α was obtained using the *chi2kink* method.^60^ The STM tip parameters were fitted from tunneling spectra measured on bare Pb surface separately for each data set. Additional details on the deconvolution procedure are presented in the Supporting Note 2. The modified ana_cont code is available from the authors upon request.

### Definition of the Kondo Temperature

The Kondo temperature *T*_K_ of the single impurity Anderson model (see Supporting Note 3) at half-filling (ϵ = −*U*/2) reads^61^
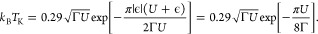
1The value of the numerical prefactor depends on the definition of *T*_K_. We use Wilson’s definition for the wide-band limit as it is common in NRG, perturbation theory, and Bethe ansatz studies. A discussion of how this definition relates to other commonly used ones and a useful table of conversions between different Kondo temperature definitions can be found in the literature.^62,63^ For our purposes, one only needs to relate the above definition of *T*_K_ to the Frota parameter Γ_F_. This parameter can be extracted from the normal (nonsuperconducting) state zero energy Kondo anomaly by fitting the peak with the standard Frota formula^30,64^
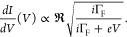
2The parameter Γ_F_ is tied to the half-width taken at half-maximum Γ_HWHM_ of the peak by Γ_HWHM_ = 2.542Γ_F_. In experiments Γ_HWHM_ is often synonymous with the Kondo temperature *T*_KF_ if *T*_KF_ ≫ *T*_exp_ or is extracted from the Fermi liquid result,
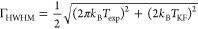
3if the experimental temperature *k*_*B*_*T*_exp_ is comparable to the width of the Kondo peak. However, Wilson’s *T*_K_ is related to Γ_HWHM_ by *k*_B_*T*_K_ = Γ_HWHM_/3.7 and consequently to the Frota fit by *k*_B_*T*_K_ = 0.686 Γ_F_ if *T*_exp_ is small. For the sake of clarity, the symbol *T*_K_^FF^ is used for Wilson’s *T*_K_ extracted by the Frota fit. This should not be mistaken for *T*_KF_ as *T*_K_^FF^ = *T*_KF_/3.7.

### NRG Calculations

All NRG calculations were performed using the open source NRG Ljubljana package.^44^ The half-bandwidth of the conduction band was fixed to 1 eV and the superconducting gap to Δ = 1.31 meV. The charging energy was set to *U* = 200 meV, as a similar value was suggested by previous studies of TBTAP molecules on surfaces^25,26^ and, unless stated otherwise, the local energy level to ϵ = −*U*/2 which corresponds to a half-filled orbital. For the sake of reproducibility, we state here the NRG Ljubjana parameters used in our calculations. Detailed explanations of these parameters can be found in the package manual.^44^ Single channel calculations of the subgap states for the case of a single molecule and the dimer system with ζ = 1, where obtained for λ = 2, symtype = SPSU2, keepenergy = 10, keep = 6000, keepmin = 2000. For equivalent double-channel calculations we used λ = 4, keepenergy = 6, keep = 5000, keepmin = 1200. The impurity spectral functions have been obtained utilizing the *z*-averaging with *N_z_* = 8 and using the modified log-Gaussian kernel broadening (smooth = newsc) which allowed setting different values of the broadening within the superconducting gap (omega0 = 1e-4) and above it (α = 0.15).

## Data Availability

The data supporting the findings of this study are available from Zenodo^65^ and the corresponding authors upon request.

## Supporting Information Available

Grid spectroscopy of individual molecules; deconvolution of tunneling spectra; superconducting single impurity Anderson model; Kondo temperature in normal state; relation between Kondo temperature and scanning tip height; superconducting two impurity Anderson model; additional results on molecular dimers; wide energy range tunneling spectra on molecular trimer (PDF)

## Notes

A previous version of this manuscript has been deposited on a preprint server.^66^

## References

[ref1] Heinrich B. W.; Pascual J. I.; Franke K. J. Single Magnetic Adsorbates on s-Wave Superconductors. Prog. Surf. Sci. 2018, 93, 1–19. 10.1016/j.progsurf.2018.01.001.

[ref2] Linder J.; Robinson J. W. Superconducting Spintronics. Nat. Phys. 2015, 11, 307–315. 10.1038/nphys3242.

[ref3] Nadj-Perge S.; Drozdov I. K.; Li J.; Chen H.; Jeon S.; Seo J.; MacDonald A. H.; Bernevig B. A.; Yazdani A. Observation of Majorana Fermions in Ferromagnetic Atomic Chains on a Superconductor. Science 2014, 346, 602–607. 10.1126/science.1259327. 25278507

[ref4] Trahms M.; Melischek L.; Steiner J. F.; Mahendru B.; Tamir I.; Bogdanoff N.; Peters O.; Reecht G.; Winkelmann C. B.; von Oppen F.; Franke K. J. Diode Effect in Josephson Junctions With a Single Magnetic Atom. Nature 2023, 615, 628–633. 10.1038/s41586-023-05743-z. 36890238 PMC10033399

[ref5] Balatsky A. V.; Vekhter I.; Zhu J.-X. Impurity-Induced States in Conventional and Unconventional Superconductors. Rev. Mod. Phys. 2006, 78, 373–433. 10.1103/RevModPhys.78.373.

[ref6] Ruby M.; Peng Y.; von Oppen F.; Heinrich B. W.; Franke K. J. Orbital Picture of Yu-Shiba-Rusinov Multiplets. Phys. Rev. Lett. 2016, 117, 186801 10.1103/PhysRevLett.117.186801. 27835014

[ref7] Choi D.-J.; Rubio-Verdú C.; de Bruijckere J.; Ugeda M. M.; Lorente N.; Pascual J. I. Mapping the Orbital Structure of Impurity Bound States in a Superconductor. Nat. Commun. 2017, 8, 15175 10.1038/ncomms15175. 28480879 PMC5424157

[ref8] Cornils L.; Kamlapure A.; Zhou L.; Pradhan S.; Khajetoorians A.; Fransson J.; Wiebe J.; Wiesendanger R. Spin-Resolved Spectroscopy of the Yu-Shiba-Rusinov States of Individual Atoms. Phys. Rev. Lett. 2017, 119, 197002 10.1103/PhysRevLett.119.197002. 29219531

[ref9] Kim H.; Palacio-Morales A.; Posske T.; Rózsa L.; Palotás K.; Szunyogh L.; Thorwart M.; Wiesendanger R. Toward Tailoring Majorana Bound States in Artificially Constructed Magnetic Atom Chains on Elemental Superconductors. Sci. Adv. 2018, 4, eaar5251 10.1126/sciadv.aar5251. 29756034 PMC5947976

[ref10] Odobesko A.; Di Sante D.; Kowalski A.; Wilfert S.; Friedrich F.; Thomale R.; Sangiovanni G.; Bode M. Observation of Tunable Single-Atom Yu-Shiba-Rusinov States. Phys. Rev. B 2020, 102, 174504 10.1103/PhysRevB.102.174504.

[ref11] Liebhaber E.; Rütten L. M.; Reecht G.; Steiner J. F.; Rohlf S.; Rossnagel K.; von Oppen F.; Franke K. J. Quantum Spins and Hybridization in Artificially-Constructed Chains of Magnetic Adatoms on a Superconductor. Nat. Commun. 2022, 13, 2160 10.1038/s41467-022-29879-0. 35443753 PMC9021194

[ref12] Kezilebieke S.; Dvorak M.; Ojanen T.; Liljeroth P. Coupled Yu-Shiba-Rusinov States in Molecular Dimers on NbSe_2_. Nano Lett. 2018, 18, 2311–2315. 10.1021/acs.nanolett.7b05050. 29533636 PMC6095633

[ref13] Malavolti L.; Briganti M.; Hänze M.; Serrano G.; Cimatti I.; McMurtrie G.; Otero E.; Ohresser P.; Totti F.; Mannini M.; et al. Tunable Spin-Superconductor Coupling of Spin 1/2 Vanadyl Phthalocyanine Molecules. Nano Lett. 2018, 18, 7955–7961. 10.1021/acs.nanolett.8b03921. 30452271

[ref14] Shahed S. M. F.; Ara F.; Hossain M. I.; Katoh K.; Yamashita M.; Komeda T. Observation of Yu-Shiba-Rusinov States and Inelastic Tunneling Spectroscopy for Intramolecule Magnetic Exchange Interaction Energy of Terbium Phthalocyanine (TbPc) Species Adsorbed on Superconductor NbSe_2_. ACS Nano 2022, 16, 7651–7661. 10.1021/acsnano.1c11221. 35467334 PMC9134493

[ref15] Xia H.-N.; Minamitani E.; Žitko R.; Liu Z.-Y.; Liao X.; Cai M.; Ling Z.-H.; Zhang W.-H.; Klyatskaya S.; Ruben M.; Fu Y.-S. Spin-Orbital Yu-Shiba-Rusinov States in Single Kondo Molecular Magnet. Nat. Commun. 2022, 13, 6388 10.1038/s41467-022-34187-8. 36302772 PMC9613647

[ref16] Homberg J.; Weismann A.; Berndt R.; Gruber M. Inducing Controlling Molecular Magnetism Through Supramolecular Manipulation. ACS Nano 2020, 14, 17387–17395. 10.1021/acsnano.0c07574. 33225694

[ref17] Li C.; Homberg J.; Weismann A.; Berndt R. On-Surface Synthesis and Spectroscopy of Aluminum Phthalocyanine on Superconducting Lead. ACS Nano 2022, 16, 16987–16995. 10.1021/acsnano.2c07106. 36153959

[ref18] Huang H.; Drost R.; Senkpiel J.; Padurariu C.; Kubala B.; Yeyati A. L.; Cuevas J. C.; Ankerhold J.; Kern K.; Ast C. R. Quantum Phase Transitions and the Role of Impurity-Substrate Hybridization in Yu-Shiba-Rusinov States. Commun. Phys. 2020, 3, 199 10.1038/s42005-020-00469-0.

[ref19] Karan S.; Huang H.; Ivanovic A.; Padurariu C.; Kubala B.; Kern K.; Ankerhold J.; Ast C. R. Tracking a Spin-Polarized Superconducting Bound State Across a Quantum Phase Transition. Nat. Commun. 2024, 15, 459 10.1038/s41467-024-44708-2. 38212303 PMC10784290

[ref20] Chatzopoulos D.; Cho D.; Bastiaans K. M.; Steffensen G. O.; Bouwmeester D.; Akbari A.; Gu G.; Paaske J.; Andersen B. M.; Allan M. P. Spatially Dispersing Yu-Shiba-Rusinov States in the Unconventional Superconductor FeTe_0.55_Se_0.45_. Nat. Commun. 2021, 12, 298 10.1038/s41467-020-20529-x. 33436594 PMC7804303

[ref21] Mas-Torrent M.; Crivillers N.; Rovira C.; Veciana J. Attaching Persistent Organic Free Radicals to Surfaces: How and Why. Chem. Rev. 2012, 112, 2506–2527. 10.1021/cr200233g. 22188381

[ref22] Zhang Y.-h.; Kahle S.; Herden T.; Stroh C.; Mayor M.; Schlickum U.; Ternes M.; Wahl P.; Kern K. Temperature and Magnetic Field Dependence of a Kondo System in the Weak Coupling Regime. Nat. Commun. 2013, 4, 2110 10.1038/ncomms3110. 23817525 PMC3730050

[ref23] Sun Q.; Mateo L. M.; Robles R.; Ruffieux P.; Lorente N.; Bottari G.; Torres T.; Fasel R. Inducing Open-Shell Character in Porphyrins Through Surface-Assisted Phenalenyl π-Extension. J. Am. Chem. Soc. 2020, 142, 18109–18117. 10.1021/jacs.0c07781. 32985889

[ref24] He Y.; Li N.; Castelli I. E.; et al. Observation of Biradical Spin Coupling through Hydrogen Bonds. Phys. Rev. Lett. 2022, 128, 236401 10.1103/PhysRevLett.128.236401. 35749188

[ref25] Li C.; Kaspar C.; Zhou P.; Liu J.-C.; Chahib O.; Glatzel T.; Häner R.; Aschauer U.; Decurtins S.; Liu S.-X.; Thoss M.; Meyer E.; Pawlak R. Strong Signature of Electron-Vibration Coupling in Molecules on Ag(111) Triggered by Tip-Gated Discharging. Nat. Commun. 2023, 14, 5956 10.1038/s41467-023-41601-2. 37749099 PMC10519934

[ref26] Pawlak R. Gate-Tunable Topological Superconductivity in a Supramolecular Electron Spin Lattice. arXiv (Condensed Matter) 22 December 2023, 2310.18134, Ver.2., https://arxiv.org/abs/2310.18134 (accessed 20 January 2024).

[ref27] Dominikowska J. Halogen-Bonded Haloamine Trimers-Modelling the X_3_ Synthon. Phys. Chem. Chem. Phys. 2020, 22, 21938–21946. 10.1039/D0CP03352A. 32974627

[ref28] Peyrot D.; Silly M. G.; Silly F. X_3_ Synthon Geometries in Two-Dimensional Halogen-Bonded 1,3,5-tris(3,5-dibromophenyl)benzene Self-Assembled Nanoarchitectures on Au(111)-(22×√3). Phys. Chem. Chem. Phys. 2018, 20, 3918–3924. 10.1039/C7CP06488H. 29318234

[ref29] Yang Z.; Fromm L.; Sander T.; Gebhardt J.; Schaub T. A.; Görling A.; Kivala M.; Maier S. On-Surface Assembly of Hydrogen- and Halogen-Bonded Supramolecular Graphyne-Like Networks. Angew. Chem., Int. Ed. 2020, 59, 9549–9555. 10.1002/anie.201916708. PMC7318139 32126147

[ref30] Frota H. O. Shape of the Kondo Resonance. Phys. Rev. B 1992, 45, 1096–1099. 10.1103/PhysRevB.45.1096. 10001582

[ref31] Ruby M.; Pientka F.; Peng Y.; von Oppen F.; Heinrich B. W.; Franke K. J. Tunneling Processes into Localized Subgap States in Superconductors. Phys. Rev. Lett. 2015, 115, 087001 10.1103/PhysRevLett.115.087001. 26340200

[ref32] De Franceschi S.; Kouwenhoven L.; Schönenberger C.; Wernsdorfer W. Hybrid Superconductor-Quantum Dot Devices. Nat. Nanotechnol. 2010, 5, 703–711. 10.1038/nnano.2010.173. 20852639

[ref33] Žonda M.; Pokorný V.; Janiš V.; Novotný T. Perturbation Theory of a Superconducting 0−π Impurity Quantum Phase Transition. Sci. Rep. 2015, 5, 8821 10.1038/srep08821. 25744137 PMC4351544

[ref34] Ménard G. C.; Guissart S.; Brun C.; Pons S.; Stolyarov V. S.; Debontridder F.; Leclerc M. V.; Janod E.; Cario L.; Roditchev D.; Simon P.; Cren T. Coherent Long-Range Magnetic Bound States in a Superconductor. Nat. Phys. 2015, 11, 1013–1016. 10.1038/nphys3508.

[ref35] Franke K. J.; Schulze G.; Pascual J. I. Competition of Superconducting Phenomena and Kondo Screening at the Nanoscale. Science 2011, 332, 940–944. 10.1126/science.1202204. 21596987

[ref36] Farinacci L.; Ahmadi G.; Reecht G.; Ruby M.; Bogdanoff N.; Peters O.; Heinrich B. W.; von Oppen F.; Franke K. J. Tuning the Coupling of an Individual Magnetic Impurity to a Superconductor: Quantum Phase Transition and Transport. Phys. Rev. Lett. 2018, 121, 196803 10.1103/PhysRevLett.121.196803. 30468615

[ref37] Roch N.; Florens S.; Bouchiat V.; Wernsdorfer W.; Balestro F. Quantum Phase Transition in a Single-Molecule Quantum Dot. Nature 2008, 453, 633–637. 10.1038/nature06930. 18509439

[ref38] Esat T.; Lechtenberg B.; Deilmann T.; Wagner C.; Krüger P.; Temirov R.; Rohlfing M.; Anders F. B.; Tautz F. S. A Chemically Driven Quantum Phase Transition in a Two-Molecule Kondo System. Nat. Phys. 2016, 12, 867–873. 10.1038/nphys3737.

[ref39] Serwatka T.; Melko R. G.; Burkov A.; Roy P.-N. Quantum Phase Transition in the One-Dimensional Water Chain. Phys. Rev. Lett. 2023, 130, 026201 10.1103/PhysRevLett.130.026201. 36706406

[ref40] Wang F.; Shen W.; Shui Y.; Chen J.; Wang H.; Wang R.; Qin Y.; Wang X.; Wan J.; Zhang M.; Lu X.; Yang T.; Song F. Electrically Controlled Nonvolatile Switching of Single-Atom Magnetism in a Dy@C_84_ Single-Molecule Transistor. Nat. Commun. 2024, 15, 2450 10.1038/s41467-024-46854-z. 38503743 PMC10951203

[ref41] Meden V. The Anderson-Josephson Quantum Dot-a Theory Perspective. J. Phys.: Condens. Matter 2019, 31, 163001 10.1088/1361-648X/aafd6a. 30630142

[ref42] Luitz D. J.; Assaad F. F.; Novotný T.; Karrasch C.; Meden V. Understanding the Josephson Current Through a Kondo-Correlated Quantum Dot. Phys. Rev. Lett. 2012, 108, 227001 10.1103/PhysRevLett.108.227001. 23003641

[ref43] Kadlecová A.; Žonda M.; Pokorný V.; Novotný T. Practical Guide to Quantum Phase Transitions in Quantum-Dot-Based Tunable Josephson Junctions. Phys. Rev. Appl. 2019, 11, 044094 10.1103/PhysRevApplied.11.044094.

[ref44] Žitko R. NRG Ljubljana. 2021 10.5281/zenodo.4841076.

[ref45] Žonda M.; Stetsovych O.; Korytár R.; Ternes M.; Temirov R.; Raccanelli A.; Tautz F. S.; Jelínek P.; Novotný T.; Švec M. Resolving Ambiguity of the Kondo Temperature Determination in Mechanically Tunable Single-Molecule Kondo Systems. J. Phys. Chem. Lett. 2021, 12, 6320–6325. 10.1021/acs.jpclett.1c01544. 34228474

[ref46] Eickhoff F.; Lechtenberg B.; Anders F. B. Effective Low-Energy Description of the Two-Impurity Anderson Model: RKKY Interaction and Quantum Criticality. Phys. Rev. B 2018, 98, 115103 10.1103/PhysRevB.98.115103.

[ref47] Yao N. Y.; Moca C. P.; Weymann I.; Sau J. D.; Lukin M. D.; Demler E. A.; Zaránd G. Phase Diagram and Excitations of a Shiba Molecule. Phys. Rev. B 2014, 90, 241108 10.1103/PhysRevB.90.241108.

[ref48] Zalom P.; Wrześniewski K.; Novotný T.; Weymann I. Double Quantum Dot Andreev Molecules: Phase Diagrams and Critical Evaluation of Effective Models. Phys. Rev. B 2024, 110, 134506 10.1103/PhysRevB.110.134506.

[ref49] Žitko R.; Bonča J. Multiple-Impurity Anderson Model for Quantum Dots Coupled in Parallel. Phys. Rev. B 2006, 74, 045312 10.1103/PhysRevB.74.045312.

[ref50] Žonda M.; Zalom P.; Novotný T.; Loukeris G.; Bätge J.; Pokorný V. Generalized Atomic Limit of a Double Quantum Dot Coupled to Superconducting Leads. Phys. Rev. B 2023, 107, 115407 10.1103/PhysRevB.107.115407.

[ref51] Hoffman S.; Klinovaja J.; Meng T.; Loss D. Impurity-Induced Quantum Phase Transitions and Magnetic Order in Conventional Superconductors: Competition Between Bound and Quasiparticle States. Phys. Rev. B 2015, 92, 125422 10.1103/PhysRevB.92.125422.

[ref52] Fernández-Torrente I.; Kreikemeyer-Lorenzo D.; Stróżecka A.; Franke K. J.; Pascual J. I. Gating the Charge State of Single Molecules by Local Electric Fields. Phys. Rev. Lett. 2012, 108, 036801 10.1103/PhysRevLett.108.036801. 22400769

[ref53] Island J. O.; Gaudenzi R.; de Bruijckere J.; Burzurí E.; Franco C.; Mas-Torrent M.; Rovira C.; Veciana J.; Klapwijk T. M.; Aguado R.; van der Zant H. S. J. Proximity-Induced Shiba States in a Molecular Junction. Phys. Rev. Lett. 2017, 118, 117001 10.1103/PhysRevLett.118.117001. 28368652

[ref54] Zhou P.; Aschauer U.; Decurtins S.; Feurer T.; Häner R.; Liu S.-X. Effect of Tert-Butyl Groups on Electronic Communication Between Redox Units in Tetrathiafulvalene-Tetraazapyrene Triads. Chem. Commun. 2021, 57, 12972–12975. 10.1039/D1CC05671A. PMC8640732 34792067

[ref55] Liu J.; Li C.; Liu X.; Lu Y.; Xiang F.; Qiao X.; Cai Y.; Wang Z.; Liu S.; Wang L. Positioning and Switching Phthalocyanine Molecules on a Cu(100) Surface at Room Temperature. ACS Nano 2014, 8, 12734–12740. 10.1021/nn5058535. 25493328

[ref56] Li C.; Wang Z.; Lu Y.; Liu X.; Wang L. Conformation-Based Signal Transfer and Processing at the Single-Molecule Level. Nat. Nanotechnol. 2017, 12, 1071–1076. 10.1038/nnano.2017.179. 28920965

[ref57] Balasubramani S. G.; Chen G. P.; Coriani S.; Diedenhofen M.; Frank M. S.; Franzke Y. J.; Furche F.; Grotjahn R.; Harding M. E.; et al. TURBOMOLE: Modular Program Suite for Ab Initio Quantum-Chemical and Condensed-Matter Simulations. J. Chem. Phys. 2020, 152 (18), 184107 10.1063/5.0004635. 32414256 PMC7228783

[ref58] Jarrell M.; Gubernatis J. Bayesian Inference and the Analytic Continuation of Imaginary-Time Quantum Monte Carlo Data. Phys. Rep. 1996, 269, 133–195. 10.1016/0370-1573(95)00074-7.

[ref59] Kaufmann J.; Held K. ana_cont: Python Package for Analytic Continuation. Comput. Phys. Commun. 2023, 282, 108519 10.1016/j.cpc.2022.108519.

[ref60] Bergeron D.; Tremblay A.-M. S. Algorithms for Optimized Maximum Entropy and Diagnostic Tools for Analytic Continuation. Phys. Rev. E 2016, 94, 023303 10.1103/PhysRevE.94.023303. 27627408

[ref61] Hewson A. C. The Kondo Problem to Heavy Fermions; Cambridge Studies in Magnetism; Cambridge University Press, 1993.

[ref62] Žitko R. Kondo Resonance Lineshape of Magnetic Adatoms on Decoupling Layers. Phys. Rev. B 2011, 84, 195116 10.1103/PhysRevB.84.195116.

[ref63] Turco E.; Aapro M.; Ganguli S. C.; Krane N.; Drost R.; Sobrino N.; Bernhardt A.; Juríček M.; Fasel R.; Ruffieux P.; Liljeroth P.; Jacob D. Demonstrating Kondo Behavior by Temperature-Dependent Scanning Tunneling Spectroscopy. Phys. Rev. Res. 2024, 6, L022061 10.1103/PhysRevResearch.6.L022061.

[ref64] Frota H. O.; Oliveira L. N. Photoemission Spectroscopy for the Spin-Degenerate Anderson Model. Phys. Rev. B 1986, 33, 7871–7874. 10.1103/PhysRevB.33.7871. 9938175

[ref65] Li C. SPM Images and dI/dV Data for Nanoscale Control of Quantum States in Radical Molecules on Superconducting Pb(111). 2024 10.5281/zenodo.14052974.

[ref66] Li C.; Pokorný V.; Žonda M.; Liu J.-C.; Zhou P.; Chahib O.; Glatzel T.; Häner R.; Decurtins S.; Liu S.-X.; Pawlak R.; Meyer E. Nanoscale Control of Quantum States in Radical Molecules on Superconducting Pb(111). arXiv (Condensed Matter) 9 August 2024, 2408.05115, Ver. 1. https://arxiv.org/abs/2408.05115. (accessed December 5, 2024).

