# Supplementary material for: Individual Assembly of Radical Molecules on Superconductors: Demonstrating Quantum Spin Behavior and Bistable Charge Rearrangement
Source: ACS Nano. 2025 Jan 14;19(3):3403–13. doi: 10.1021/acsnano.4c12387 (PMC11781030; doi:10.1021/acsnano.4c12387)
Supplement: Supplementary file 1 — nn4c12387_si_001.pdf [file nn4c12387_si_001.pdf]

Supporting Information: Individual Assembly of Radical Molecules on Superconductors: Demonstrating Quantum Spin Behavior and Bistable Charge Rearrangement

Chao Li,[1,*] Vladislav Pokorný,[2] Martin Žonda,[3,†] Jung-Ching Liu,[1] Ping Zhou,[4] Outhmane Chahib,[1] Thilo Glatzel,[1] Robert Häner,[4] Silvio Decurtins,[4] Shi-Xia Liu,[4,‡] Rémy Pawlak,[1,§] and Ernst Meyer[1,¶]

[1]*Department of Physics, University of Basel, Klingelbergstrasse 82, 4056 Basel, Switzerland.*
[2]*Institute of Physics (FZU), Czech Academy of Sciences, Na Slovance 2, 182 00 Prague 8, Czech Republic.*
[3]*Department of Condensed Matter Physics, Faculty of Mathematics and Physics, Charles University, Ke Karlovu 5, 121 16 Prague 2, Czech Republic.*
[4]*Department of Chemistry, Biochemistry and Pharmaceutical Sciences, W. Inäbnit Laboratory for molecular quantum materials, University of Bern, Freiestrasse 3, 3012 Bern, Switzerland.*

SUPPLEMENTARY NOTE 1: GRID SPECTROSCOPY OF INDIVIDUAL MOLECULES

Supplementary Figure 1 provides additional details on grid spectroscopy of TBTAP molecules.

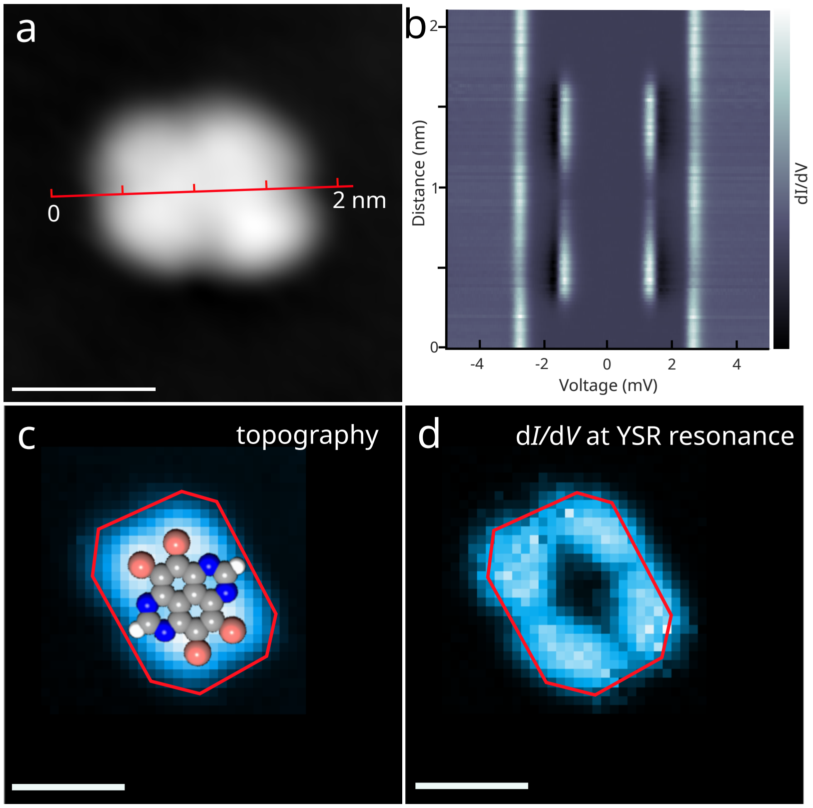

Supplementary Figure 1. (**a** - **b**) Tunneling spectra were acquired at diverse positions along the TBTAP molecule, ranging from the left side to the right side, providing a visual representation of the shape of the YSR resonances. (V = 10 mV, I = 100pA). (**c**) Topography image obtained concurrently with the tunneling spectra. (**d**) $\mathrm{d}I/\mathrm{d}V$ map at the bias corresponding to the positive YSR peak, with a scale ranging from 0.0 to 1.6 nS. The region enclosed by the red line is derived from the topographic structure image area in **a**, indicating that the energy of YSR extends only over several atomic-scale distances in space. The white scale bars represents 1 nm. (V_s = 5 mV; I_t = 0.1 nA, A_{mod} = 0.05 mV, f = 613 Hz).

SUPPLEMENTARY NOTE 2: DECONVOLUTION OF TUNNELING SPECTRA

A superconducting tip was utilized to increase the resolution of the tunneling spectra beyond the thermal limit. As a result, the measured tunneling spectra are convolutions of the sample and tip densities of states (DOS),

$$I(V) = G \int_{-\infty}^{\infty} d\omega \rho_s(\omega)\rho_t(\omega - eV)[f(\omega - eV) - f(\omega)], \tag{1}$$

where $\rho_{s/t}$ is the DOS of the sample/tip, $f(\omega)$ is the Fermi-Dirac distribution and G is a constant proportional to the square of the tunnel matrix element between the initial and final state. A deconvolution routine was utilized to obtain the DOS of the sample. For the tip, we assume the BCS DOS,

$$\rho_t(\omega) = \mathrm{sgn}(\omega)\,\mathrm{Re}\,\frac{\omega + i\gamma_t}{\sqrt{(\omega + i\gamma_t)^2 - \Delta_t^2}}, \tag{2}$$

where Δ_t is the superconducting gap of the tip and γ_t is a parameter which broadens the coherence peaks due to the finite lifetime of quasiparticles [1]. The differential conductance is given by

$$\frac{dI}{dV} = -eG \int_{-\infty}^{\infty} d\omega \rho_s(\omega) \left[\frac{\partial \rho_t(E)}{\partial E}\Big|_{E=\omega-eV} [f(\omega - eV) - f(\omega)] + \rho_t(\omega - eV) \frac{\partial f(E)}{\partial E}\Big|_{E=\omega-eV} \right]. \tag{3}$$

We estimate the values of γ_t and Δ_t from a measurement of a bare Pb(111) surface, away from the molecule. We substitute Eq. (2) for both ρ_t and ρ_s and assume that the values of γ and Δ are the same for the tip and the surface. Optimal values of the parameters can be obtained using the least-squares fitting procedure. The resulting values for different experiments are included in figure captions.

With the knowledge of ρ_t we rewrite Eq. (3) in a discretized form,

$$\left(\frac{dI}{dV}\right)_i = G \sum_{j=1}^{N_\omega} K_{ij} \rho_{s,j}, \tag{4}$$

where

$$K_{ij} = -e \left[\frac{\partial \rho_t(E)}{\partial E}\Big|_{E=\omega_j - eV_i} [f(\omega_j - eV_i) - f(\omega_j)] + \rho_t(\omega_j - eV_i) \frac{\partial f(E)}{\partial V}\Big|_{E=\omega_j - eV_i} \right] \delta\omega, \tag{5}$$

V_i, $i = 1..N_V$ and ω_j, $j = 1..N_\omega$, are discrete, equidistant voltage and energy values, $\rho_{s,j} = \rho_s(\omega_j)$ and $\delta\omega = \omega_2 - \omega_1$.

In general, such deconvolution is an ill-posed problem (the solution is not unique), since the condition number of the kernel matrix K is very large and the direct inversion of K is not possible (even for $N_V = N_\omega$). Usually, an approximate solution can be obtained using the Moore-Penrose pseudoinverse [2] or by assuming the functional form of the surface DOS and fitting its parameters [3]. A more reliable general solution can be obtained using the maximum entropy method (MEM). This approach was previously utilized for the analytic continuation of imaginary-time Green functions to real frequencies [4]. Instead of minimizing the misfit function,

$$\chi^2(\rho_s) = \left(\sum_i \left[G \sum_j K_{ij}\rho_{s,j} - \left(\frac{dI}{dV}\right)_i \right] \right)^T C^{-1} \left(\sum_i \left[G \sum_j K_{ij}\rho_{s,j} - \left(\frac{dI}{dV}\right)_i \right] \right), \tag{6}$$

where C is the covariance matrix, we minimize a regularized expression $\chi^2(\rho_s)/2 - \alpha S(\rho_s)$, where α is an ad-hoc hyperparameter that needs to be specified outside of the method,

$$S(\rho_s) = \sum_{i=1}^{N_\omega} \left[\rho_{s,i} - D_i - \rho_{s,i} \log \frac{\rho_{s,i}}{D_i} \right] \tag{7}$$

is the entropy and D is the so-called default model which contains all the prior knowledge about the surface DOS.

There are many well-optimized implementations of the MEM algorithm. We used a modified `ana_cont` package [5] in which we implemented the kernel (5). We used a flat default model of the same width as the input data which corresponds to no prior knowledge. We assumed a diagonal covariance matrix and the standard deviation of $\mathrm{d}I/\mathrm{d}V$ was guessed to 10^{-13} S. The final results depend only very weakly on this value. The optimal value of the hyperparameter α was obtained using the *chi2kink* method [6]. The resulting surface DOS is nonnegative by definition and robust with regard to noise in the input $\mathrm{d}I/\mathrm{d}V$ data. An example of the surface DOS obtained using the MEM deconvolution is plotted in Supplementary Figure 2. The modified `ana_cont` code is available from the authors upon request.

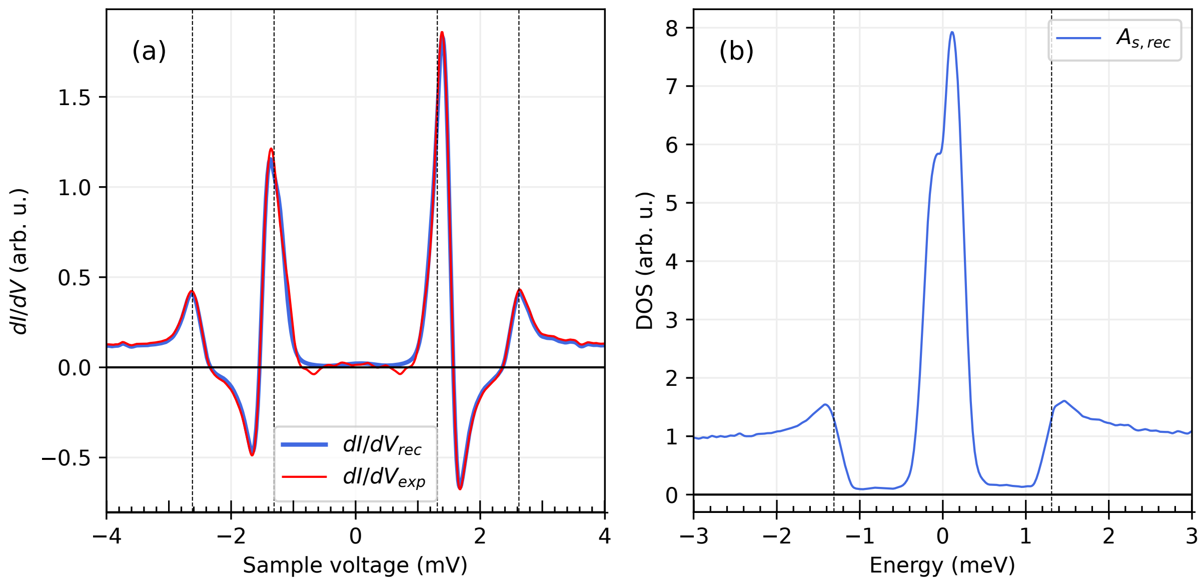

Supplementary Figure 2. (**a**) Differential conductance spectrum which corresponds to $h = 0$ in Figure 2d in the main text, measured at $T_{\text{exp}} = 1\,\text{K}$. Red line represents the experimental result, blue line the spectrum reconstructed from the deconvolved surface DOS to check the quality of the procedure. The vertical dashed lines mark the positions of the gap edges $\pm\Delta$ and the doubled gap edges $\pm 2\Delta$. (**b**) The deconvolved surface DOS corresponding to the $\mathrm{d}I/\mathrm{d}V$ data in panel **a**. The parameters of the tip read $\Delta_{\text{t}} = 1.31\,\text{meV}$ and $\gamma_{\text{t}} = 0.04\,\text{meV}$.

SUPPLEMENTARY NOTE 3: SUPERCONDUCTING SINGLE IMPURITY ANDERSON MODEL

The spectral properties of the investigated spin one-half molecule on a superconducting surface can be modeled using the superconducting single impurity Anderson model (SC-SIAM) [7]. This model describes a quantum impurity coupled to two superconducting leads, i.e. the surface (s) and the tip (t). Its general Hamiltonian can be written as

$$\mathcal{H} = \mathcal{H}_{1\text{d}}^{\text{imp}} + \sum_{j=\text{s,t}} \left(\mathcal{H}_j^{\text{lead}} + \mathcal{H}_j^{\text{hyb}} \right), \tag{8}$$

where $\mathcal{H}_{1\text{d}}^{\text{imp}}$ describes the impurity and reads

$$\begin{aligned} \mathcal{H}_{1\text{d}}^{\text{imp}} &= \epsilon \sum_{\sigma} d_\sigma^\dagger d_\sigma + U d_\uparrow^\dagger d_\uparrow d_\downarrow^\dagger d_\downarrow \\ &= \varepsilon \sum_{\sigma} \left(d_\sigma^\dagger d_\sigma - \frac{1}{2} \right) + \frac{U}{2} \left(d_\uparrow^\dagger d_\uparrow + d_\downarrow^\dagger d_\downarrow - 1 \right)^2 + \text{const.}, \end{aligned} \tag{9}$$

where $d_\sigma^\dagger$ creates an electron with spin σ on the impurity with energy ϵ and U is the local Coulomb interaction (charging energy) on the impurity. For simplicity, we assume in our analysis the energy level $\epsilon = -U/2$ respectively $\varepsilon = 0$ that corresponds to a half-filled orbital.

The second term in Hamiltonian (8) describes the superconducting leads, i.e., the surface and the tip, within the BCS theory,

$$\mathcal{H}_j^{\text{lead}} = \sum_{\mathbf{k}\sigma} \varepsilon_{j\mathbf{k}} c_{j\mathbf{k}\sigma}^\dagger c_{j\mathbf{k}\sigma} - \Delta_j \sum_{\mathbf{k}} \left(c_{j\mathbf{k}\uparrow}^\dagger c_{j-\mathbf{k}\downarrow}^\dagger + \text{H.c.} \right), \tag{10}$$

where $c_{j\mathbf{k}\sigma}^\dagger$ creates an electron with spin σ and energy $\varepsilon_{j\mathbf{k}}$ in the lead $j \in s,t$ and Δ_j is the superconducting order parameter. In what follows, we assume $\varepsilon_{s\mathbf{k}} = \varepsilon_{t\mathbf{k}}$ and $\Delta_s = \Delta_t \equiv \Delta$ as the surface and the tip are made from the same material. For $\Delta = 0$ we obtain the standard SIAM with metallic lead.

The last term in Hamiltonian (8) describes the hybridization between the impurity and the leads,

$$\mathcal{H}_j^{\text{hyb}} = \sum_{\mathbf{k}\sigma} \left(V_{j\mathbf{k}} c_{j\mathbf{k}\sigma}^\dagger d_\sigma + \text{H.c.} \right), \tag{11}$$

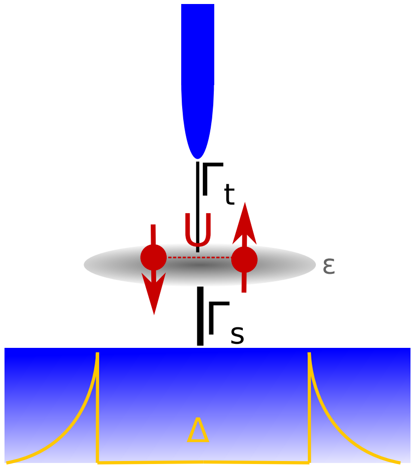

Supplementary Figure 3. Illustration of the SC-SIAM where Γ_t (Γ_s) are constant tunneling rates between the impurity and the tip (substrate). We assume $\Gamma_s \gg \Gamma_t$. Parameter U is the strength of the repulsive Coulomb interaction between the electrons on the impurity and ε the impurity energy level. Both the substrate and the tip are BCS superconductors with equal superconducting gap Δ.

where $V_{j\mathbf{k}}$ is a tunneling matrix element between the lead $j =$ s,t and the impurity. In our analysis, we assume the tunnel coupling magnitudes $\Gamma_j(E) = \pi \sum_{\mathbf{k}} |V_{j\mathbf{k}}|^2 \delta(E - \varepsilon_{j\mathbf{k}})$ to be constant. The total coupling of the impurity to the leads is then $\Gamma = \Gamma_\mathrm{s} + \Gamma_\mathrm{t}$.

SUPPLEMENTARY NOTE 4: KONDO TEMPERATURE IN NORMAL STATE

Supplementary Figure 4a shows the evolution of the tunneling spectrum of TBTAP with increasing magnetic field taken at the position marked by the black dot in panel **b** at temperature $T_{exp} = 2.2\,\mathrm{K}$. At $1\,\mathrm{T}$ the superconductivity is completely suppressed in both the substrate and the tip. This tunneling spectrum was fitted using the Frota formula (Eq. 2 in the main text; panel **c**). The Frota fit gives the Frota parameter $\Gamma_\mathrm{F} \doteq 0.952\,\mathrm{meV}$. The half-width of the peak then reads $\Gamma_\mathrm{HWHM} \doteq 2.42\,\mathrm{meV}$ and the estimated Kondo temperature is $T_\mathrm{K}^\mathrm{FF} = 7.5\,\mathrm{K}$. While the exact tip height above the molecule during the Kondo peak measurement is unknown, we compared the normal-state conductance (conductance at large voltage) with the results from the QPT measurement. Based on this comparison (not shown), the Frota fit was applied at a tip height close to that at which the QPT occurs. The spatial map of the Kondo peak is plotted in Supplementary Figure 4d. Its shape is identical to the shape of the YSR state and has the same symmetry as the spin density of the molecule calculated using DFT (Figs. 2a-b in the main text).

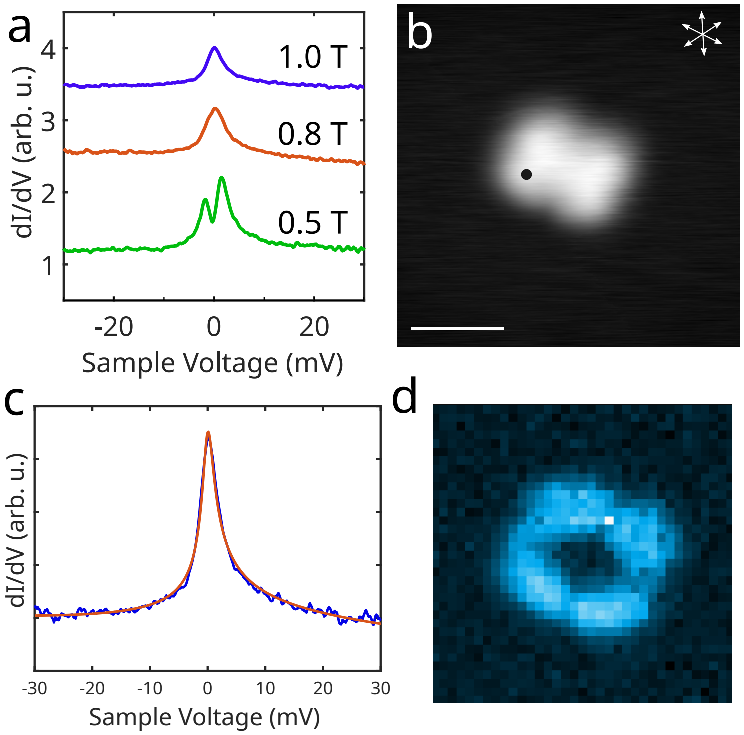

Supplementary Figure 4. (**a**) Differential conductance spectra obtained at the position indicated by a black dot in **b** at temperature of 2.2 K, spanning a range of magnetic fields from 0.5 T to 1 T. At a magnetic field of 1 T, the Kondo resonance appears due to the complete suppression of the superconductivity in both the Pb(111) surface and the Pb tip. The scale bar represents 1 nm. (**c**) The differential conductance spectra of Kondo resonance at 1 T (blue) and the Frota fit (Eq. 2 in the main text; red). The Frota fit yields an estimated Kondo temperature $T_K^{FF} = 7.5$ K. (**d**) A dI/dV map at the bias corresponding to the Kondo peak (0 V) is the same as the distribution of YSR states in the molecule (Figure 2a in the main text). The white scale bar in panel **b** represents 1 nm. ($V_s = 30$ mV; $I_t = 0.1$ nA, $A_{mod} = 0.03$ mV, $f = 613$ Hz).

SUPPLEMENTARY NOTE 5: RELATION BETWEEN KONDO TEMPERATURE AND SCANNING TIP HEIGHT

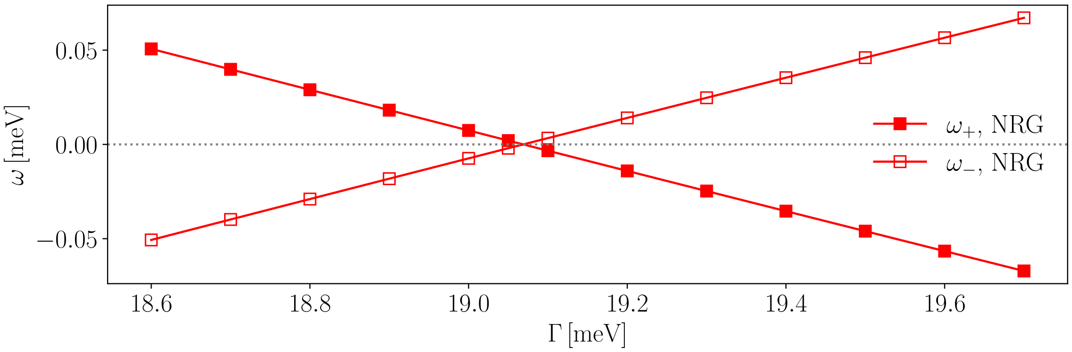

Supplementary Figure 5. Evolution of YSR state energy of SC-SIAM with respect to total coupling $\Gamma = \Gamma_s + \Gamma_t$ calculated using NRG. The model parameters are $U = 200$ meV, $\Delta = 1.31$ meV, $W = 1$ eV and $\varepsilon = 0$.

To perform a fit of the experimental YSR energy by NRG, we need the mapping between the Kondo temperature T_K (or equivalently, the total coupling Γ) and the height (z-position) of the scanning tip h.

Supplementary Figure 5 shows the NRG result on the evolution of YSR states in the vicinity of the QPT for a

window of YSR energies ω reflecting the experimental values. We use model parameters $U = 200\,\text{meV}$, $\Delta = 1.31\,\text{meV}$, $W = 1\,\text{eV}$ and $\varepsilon = 0$ and plot $\omega_\pm$ as a function of the total coupling Γ. The dependence of $\omega_\pm$ on Γ is almost perfectly linear here and can be fitted by $\omega_\pm^{\text{NRG}} = \pm(a + b\Gamma^{\text{NRG}})$ with $a \doteq 2.06$ meV and $b \doteq -108$. In principle, if all other parameters are fixed, we can extract the relation between the total coupling Γ and the tip height h as

$$\Gamma^{\text{exp}}(h) = (\omega_a^{\text{exp}} - a)/b \tag{12}$$

and use it for the mapping. However, there is an ambiguity here. Due to the symmetric nature of $\pm\omega_a$ it is not immediately clear whether larger h means stronger or weaker Γ. As argued before [8], the STM tip exerts a force on the molecule that reflects a Lennard-Jones-like potential. The force is attractive at large tip-molecule distances and repulsive for short ones. As we lower the tip, the Γ_t increases; however, the tip also lifts the molecule from the surface which decreases Γ_s. Because $\Gamma_\text{t} \ll \Gamma_\text{s}$ the net effect is a decrease in the total coupling Γ. It is expected that Γ_s changes exponentially with the distance [9] from the surface $z(h)$, which is a function of tip position h. Considering the narrow window of relevant values of Γ shown in Supplementary Figure 5 and neglecting the changes is Γ_t we can make a following linear approximation

$$\Gamma(h) = \Gamma_{z=0} \exp[-\alpha z(h)] \approx \Gamma_0 + \tilde{\alpha} h, \tag{13}$$

where the total coupling at $h = 0$ denoted Γ_0 and parameter $\tilde{\alpha}$ can be extracted from a fit using $\Gamma^{\text{exp}}(h)$ from Eq. (12). This gives us $\Gamma_0 \doteq 19.7$ meV and $\tilde{\alpha} \doteq 5.6\,\mu\text{eV/pm}$. The resulting mapping to distance h is then simply

$$h^{\text{NRG}} = (\Gamma^{\text{NRG}} - \Gamma_0)/\tilde{\alpha}. \tag{14}$$

This simple linearization is valid for $h \geq -140\,\text{pm}$ as visible from Figure 2h in the main text. For smaller distances, the potential becomes repulsive and the molecule is pushed back to the surface. Consequently, Γ starts to grow again.

SUPPLEMENTARY NOTE 6: SUPERCONDUCTING TWO IMPURITY ANDERSON MODEL

We analyze the scenario of two molecules on a substrate using the superconducting two impurity Anderson model (SC-TIAM) given by Hamiltonian

$$\mathcal{H} = \mathcal{H}_{2\mathrm{d}}^{\mathrm{imp}} + \mathcal{H}^{\mathrm{lead}} + \sum_{\ell=1,2} \mathcal{H}_{\ell}^{\mathrm{hyb}}. \tag{15}$$

The term $\mathcal{H}_{2\mathrm{d}}^{\mathrm{imp}}$ describes two interacting impurities

$$\mathcal{H}_{2\mathrm{d}}^{\mathrm{imp}} = \sum_{\ell=1,2} \left[\varepsilon_\ell \sum_\sigma \left(d_{\ell\sigma}^\dagger d_{\ell\sigma} - \frac{1}{2} \right) + \frac{U_\ell}{2} \left(d_{\ell\uparrow}^\dagger d_{\ell\uparrow} + d_{\ell\downarrow}^\dagger d_{\ell\downarrow} - 1 \right)^2 \right]$$
$$-t \sum_\sigma \left(d_{1\sigma}^\dagger d_{2\sigma} + \mathrm{H.c.} \right) + W \sum_\sigma d_{1\sigma}^\dagger d_{1\sigma} \sum_\sigma d_{2\sigma}^\dagger d_{2\sigma},$$

where $d_{\ell\sigma}^\dagger$ creates an electron with spin σ on the impurity ℓ with energy $\epsilon_\ell = \varepsilon_\ell - U_\ell/2$. We have found that the first line of terms in Eq. (16) is already sufficient for a qualitative explanation of the experiments. However, the interactions in the second line can be significant if the molecules are close to each other. In particular, we assume either a direct hopping (see Ref. [10]) of an electron between the impurities parameterized by t or an inter-molecular density-density interaction with strength W.

The second term in Hamiltonian (15) describes the superconducting substrate,

$$\mathcal{H}^{\mathrm{lead}} = \sum_{\mathbf{k}\sigma} \varepsilon_{\mathbf{k}} c_{\mathbf{k}\sigma}^\dagger c_{\mathbf{k}\sigma} - \Delta \sum_{\mathbf{k}} \left(c_{\mathbf{k}\uparrow}^\dagger c_{-\mathbf{k}\downarrow}^\dagger + \mathrm{H.c.} \right). \tag{16}$$

The last term in Hamiltonian (15) describes the hybridization between the impurity ℓ and the substrate,

$$\mathcal{H}_\ell^{\mathrm{hyb}} = \sum_{\mathbf{k}\sigma} V_{\ell\mathbf{k}} \left(c_{\mathbf{k}\sigma}^\dagger d_{\ell\sigma} + \mathrm{H.c.} \right). \tag{17}$$

Note that in general the effect of the distance between the molecules is encoded in the complex hybridization terms $V_{\ell\mathbf{k}}$ and it also affects t. However, we assume the tunnel-coupling magnitudes $\Gamma_\ell(E) = \pi \sum_{\mathbf{k}} |V_{\ell\mathbf{k}}|^2 \delta(E - \varepsilon_{\mathbf{k}}) = \Gamma_\ell$ to be constant and, therefore, for the constant DOS in the substrate, the effect of the distance on hybridization is parameterized by the cross terms of the form $\Gamma_{12}(E) = \pi \sum_{\mathbf{k}} V_{1\mathbf{k}}^* V_{2\mathbf{k}} \delta(E - \varepsilon_{\mathbf{k}}) = \zeta \sqrt{\Gamma_1 \Gamma_2}$, where $0 \le \zeta \le 1$. Equivalently, due to the additive nature of the relevant tunneling self-energies, we can model such a system by assuming two leads as illustrated in Supplementary Figure 6. The first impurity is coupled to the first lead by $(1-\delta)\Gamma_1$ and to the second lead by $\delta\Gamma_1$ and vice versa. The governing parameter $\delta \in \langle 0, 0.5 \rangle$ is related to ζ by $\zeta = 2\sqrt{(1-\delta)\delta}$. Here, $\zeta = 0$ ($\delta = 0$) models a situation in which molecules are not correlated through the substrate and, therefore, are effectively coupled to their own distinct reservoirs. The other limit $\zeta = 1$ ($\delta = 0.5$) describes a local two-level impurity coupled to the same superconducting substrate. This means that the parameter ζ can be used to tune the inter-molecular correlations that can lead to both effective coupling as well as the effective exchange interaction of Ruderman-Kittel-Kasuya-Yosida (RKKY) type. The related spin-spin correlation function $\langle \boldsymbol{S}_1 \boldsymbol{S}_2 \rangle$ of the ground state can be calculated using $\boldsymbol{S}_\ell = \frac{\hbar}{2} d_\ell^\dagger \boldsymbol{\sigma} d_\ell$ where $d_\ell^\dagger = (d_{\ell\uparrow}^\dagger, d_{\ell\downarrow}^\dagger)$ and $\boldsymbol{\sigma}$ is a vector of Pauli matrices. An additional effect of the superconducting scanning tip can be approximated by varying the relevant Γ_ℓ.

An illustration of the spin structure of SC-TIAM in the singlet and doublet ground states is shown in the Supplementary Figure 7. For $t = 0$ and $W = 0$ the ground state changes at critical δ from *many-body*, singlet characterized by the total spin (spin quantum number) $S_{\mathrm{T}} = 0$, to *many-body* doublet with $S_{\mathrm{T}} = 1/2$ as shown in panel (a) (note that $\langle \hat{S}^2 \rangle = S(S+1)$ in Planck units). However, this does not mean that the average spins measured on the impurities are fully screened in the singlet phase or that exactly one of the spins is screened in the doublet phase. As shown in panel (c) the total average spin quantum number S_i on impurity $i = 1, 2$ is still significant, i.e., $S_i \approx 0.45$ for $\delta = 0.5$. These spins are practically uncorrelated in the singlet phase, but allow for strong ferromagnetic corelation in the doublet phase with $\langle \hat{S}_1 \hat{S}_2 \rangle \approx 0.19$ at $\delta = 0.5$ as shown in panel (b). Consequently, the total spin on the dimer also increases with δ in the dimer phase as seen from $\langle \hat{S}_{\mathrm{dimer}}^2 \rangle = \langle (\hat{S}_1 + \hat{S}_2)^2 \rangle$ in panel (c).

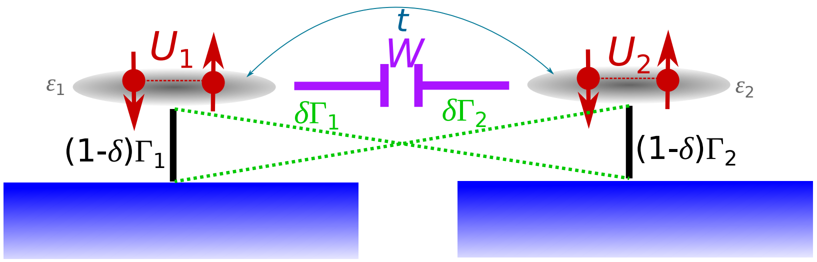

Supplementary Figure 6. Illustration of the SC-TIAM where the distance of the molecules is parameterized by $\delta \in \langle 0, 0.5\rangle$. Here $\delta = 0$ (and $t = 0$, $W = 0$) describes a situation in which molecules are decoupled (large distance). In the opposite limit $\delta = 0.5$ the model is equivalent to a single, two-level impurity coupled to a single lead as a result of the additive nature of the tunneling self-energies. If the molecules are close to each other some additional interactions can play significant role, e.g., direct hopping t and capacitive coupling W.

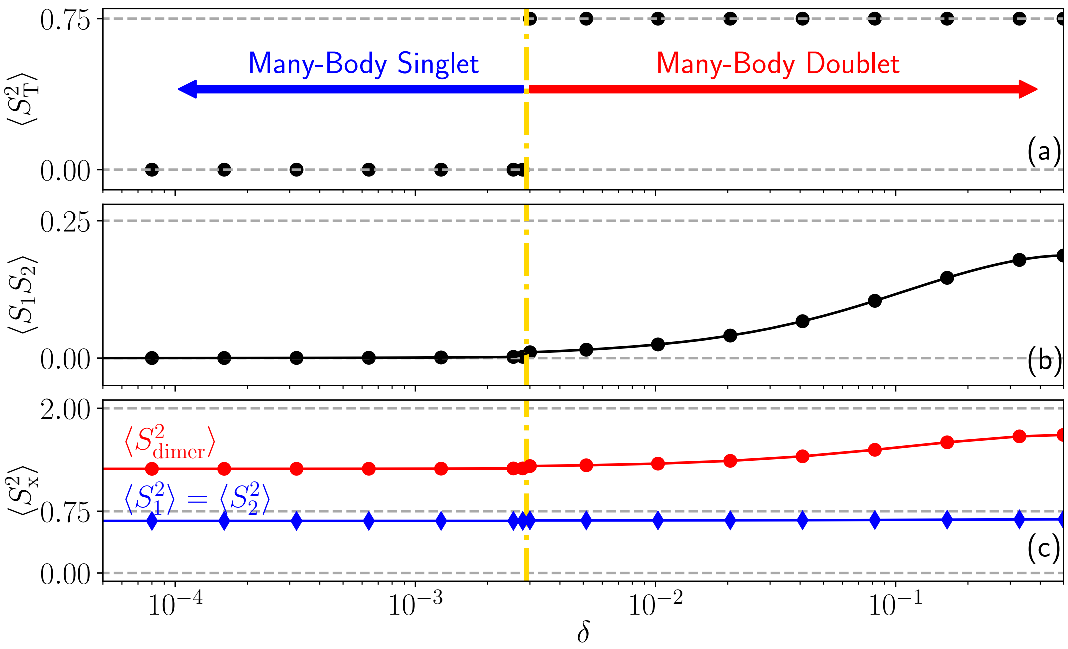

Supplementary Figure 7. Illustration of the magnetic properties of a dimer for changing parameter δ which governs the strength of the correlations through the superconducting surface. (**a**) Expectation value of the total spin squared for the many-body ground state. The vertical golden line marks the point of QPT where the total spin (spin quantum number) changes from 0 to 1/2 as $\langle \hat{S}^2\rangle = S(S+1)$ in Planck units. (**b**) Inter-impurity spin-spin correlation function. (**c**) The expectation value of the total *dimer* spin squared (red circles) and total *impurity* spin squared (blue circles). Figure demonstrates that the spins on the impurities are only partially screened which allows for strong ferromagnetic inter-impurity coupling despite the doublet many-body ground state. The model parameters used in the calculation are $U_1 = U_2 = 200\,\mathrm{meV}$, $\varepsilon_1 = \varepsilon_2 = 0$, $\Gamma_1 = \Gamma_2 = 20\,\mathrm{meV}$, and $\Delta = 1.31\,\mathrm{meV}$.

SUPPLEMENTARY NOTE 7: YSR SPECTRA OF MOLECULAR DIMERS WITH DIFFERENT GROUND STATES

Supplementary Figure 8 presents additional results on the positions of the YSR states of a molecular dimer described by SC-TIAM to complement the results in the main text. Here, one molecule is tuned to a singlet ($\Gamma_1 = 20\,\mathrm{meV}$) and the other to a doublet ($\Gamma_2 = 18.8\,\mathrm{meV}$) ground state, respectively. This change in the coupling strength can be induced by the presence of a scanning tip above one of the molecules. This contrasts with the situation in Figure 3e in the main text, where both molecules are tuned to the same singlet ground state. The combined ground state for $t = 0$ is now always a doublet. The spectra show up to three pairs of YSR states that correspond to either doublet-triplet or doublet-singlet transitions. In this way, the situation for $\zeta > 0.1$ is equivalent to the case discussed in the main

text, including the effect of nonzero direct hopping t and capacitive coupling W.

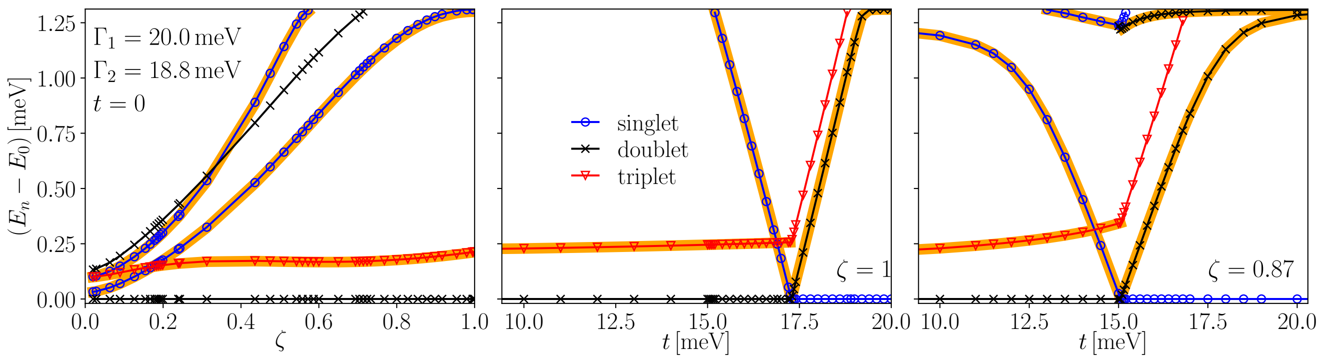

Supplementary Figure 8. YSR states of a dimer described by SC-TIAM calculated using NRG in the case of one molecule being in singlet and the other in the doublet ground state. The blue, black, and red symbols denote the singlet, doublet, and triplet states, respectively. Orange lines denote the actual YSR states, i.e., transitions which are not forbidden by the sum rule $\Delta s_z = \pm 1/2$.

SUPPLEMENTARY NOTE 8: ADDITIONAL RESULTS ON MOLECULAR DIMERS

Here, we present additional data to support our claims on molecular dimers formed by a pair of TBTAP$^{\bullet -}$ molecules. Supplementary Figure 9 shows the surface DOS obtained by the MEM deconvolution for the tunneling spectra presented in Figure 3c in the main text, together with the DOS calculated using NRG for non-zero inter-impurity (capacitive) coupling W. Such a coupling might be sizeable in the dimer, as suggested by the results on the longer molecular chains. Tuning this coupling changes the number of YSR states, as well as their position, and allows us to simulate the DOS obtained from the experimental data. This suggests that SC-TIAM as introduced in Supplementary Note 6 is a reliable description of molecular dimers. Note that the coherence peaks at $\pm\Delta$ are not visible in the calculated DOS as a result of their small weight compared to the YSR states.

Supplementary Figure 10 shows the evolution of the tunneling spectra and the surface DOS for a dimer with decreasing distance between the molecules to support the discussion of the effect of distance in the main text.

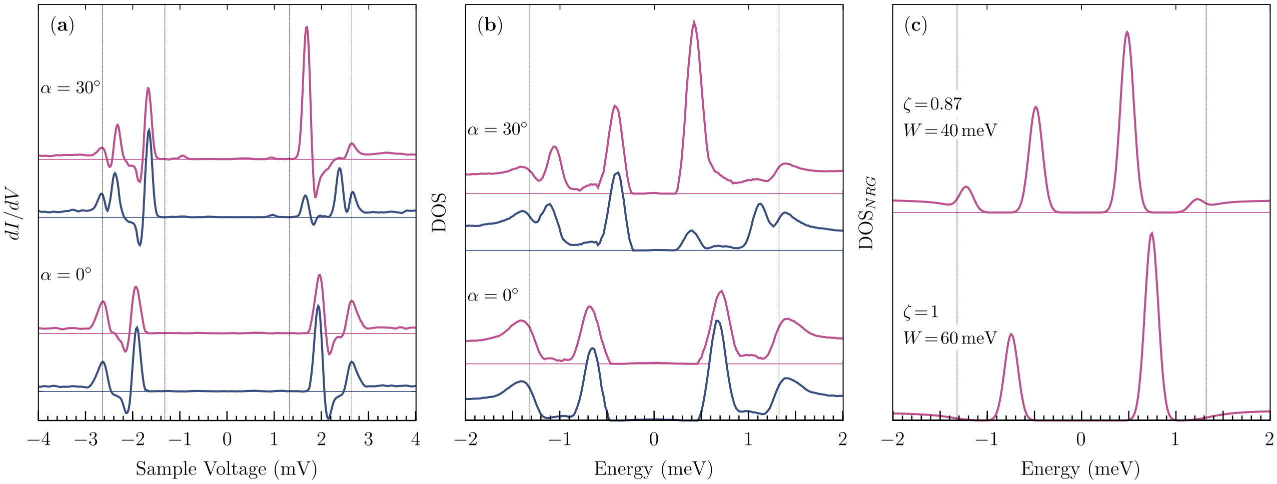

Supplementary Figure 9. (**a**) The differential conductance spectra measured using superconducting Pb tip for the dimers formed by lateral manipulation (Figure 3a-c in main text). (**b**) The surface density of states obtained using MEM deconvolution from tunneling spectra in panel **a**. The scanning tip parameters for the deconvolution read $\Delta_t = 1.32\,\mathrm{meV}$ and $\gamma_t = 0.02\,\mathrm{meV}$. (**c**) Surface DOS calculated using NRG for $U = 200\,\mathrm{meV}$, $\Gamma_1 = \Gamma_2 = 20\,\mathrm{meV}$, $t = 0$, $\zeta = 0.87$ and $W = 40\,\mathrm{meV}$ (top, $\alpha = 30°$) and $\zeta = 1$ and $W = 60\,\mathrm{meV}$ (bottom, $\alpha = 0°$), respectively. The vertical dashed lines in each panel mark the positions of $\pm\Delta$ and $\pm 2\Delta$.

SUPPLEMENTARY NOTE 9: WIDE ENERGY RANGE TUNNELING SPECTRA ON MOLECULAR TRIMER

Here, we present a detailed analysis of differential conductance spectra collected over a broad energy range for a trimer chain formed by lateral manipulation, as shown in Supplementary Figure 11. The STM image in Supplementary Figure 11a illustrates the trimer chain. The two molecules at the ends and the one at the center exhibit notably different electronic properties, attributed to their distinct charge states. Specifically, the end molecules are charged, while the central molecule remains neutral.

Supplementary Figure 11b shows the differential conductance spectra at the locations indicated by red and blue dots in panel (a). The spectra reveal a single YSR peak pair at $V_s = \pm 1.4\,\mathrm{mV}$ for the charged molecule at the end, while the central, neutral molecule exhibits only a weak YSR peak pair, originating as an extension from the signals of the charged molecules. To explore additional spectroscopic characteristics, Supplementary Figure 11c extends the energy range from -100 mV to 100 mV. Here, the charged end molecule exhibits pronounced vibrational features at ± 20, ± 30, and $\pm 50\,\mathrm{mV}$, attributed to YSR resonances [11, 12], while the neutral central molecule shows no significant spectroscopic features. Finally, Supplementary Figure 11d further extends the energy range from -300 mV to 300 mV, showing a resonance at 260 mV in the central molecule. This feature is likely associated with the lowest unoccupied molecular orbital (LUMO) of the neutral molecule, providing a clear distinction between the electronic characteristics of the charged and neutral molecules within the trimer.

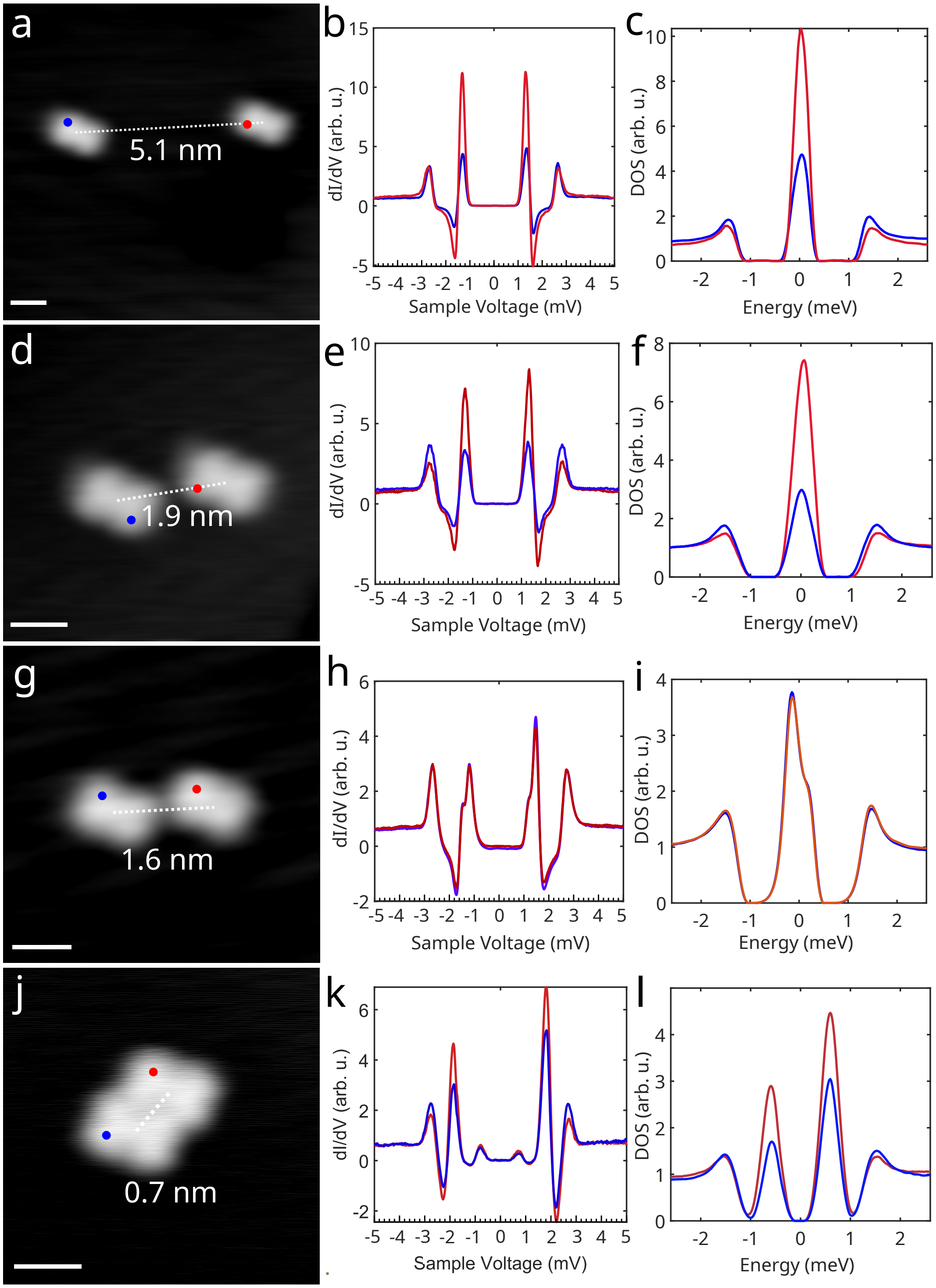

Supplementary Figure 10. (**a**, **d**, **g** and **j**) STM images of adjacent molecules in a relative distance of 5.1, 1.9, 1.6 and 0.7 nm, respectively. The white scale bar represents 1 nm. (**b**, **e**, **h** and **k**) Differential conductance spectra measured using a superconducting Pb tip. The spectra were obtained at the positions of the blue and red dots, represented by their respective colors, at temperature $T_{\mathrm{exp}} = 2.2$ K. The two smaller, low-voltage peaks in panel **k** are thermal images of the YSR states induced by the higher temperature which populates the states above the Fermi energy. The different intensities of the red/blue curves are a result of the fact that they were measured at different positions on the two molecules. (**c**, **f**, **i** and **l**) Surface DOS obtained from the $\mathrm{d}I/\mathrm{d}V$ data using the MEM deconvolution procedure. The STM tip parameters were fitted from a tunneling spectrum measured on the bare Pb surface and read $\Delta_{\mathrm{t}} = 1.32$ meV and $\gamma_{\mathrm{t}} = 0.02$ meV.

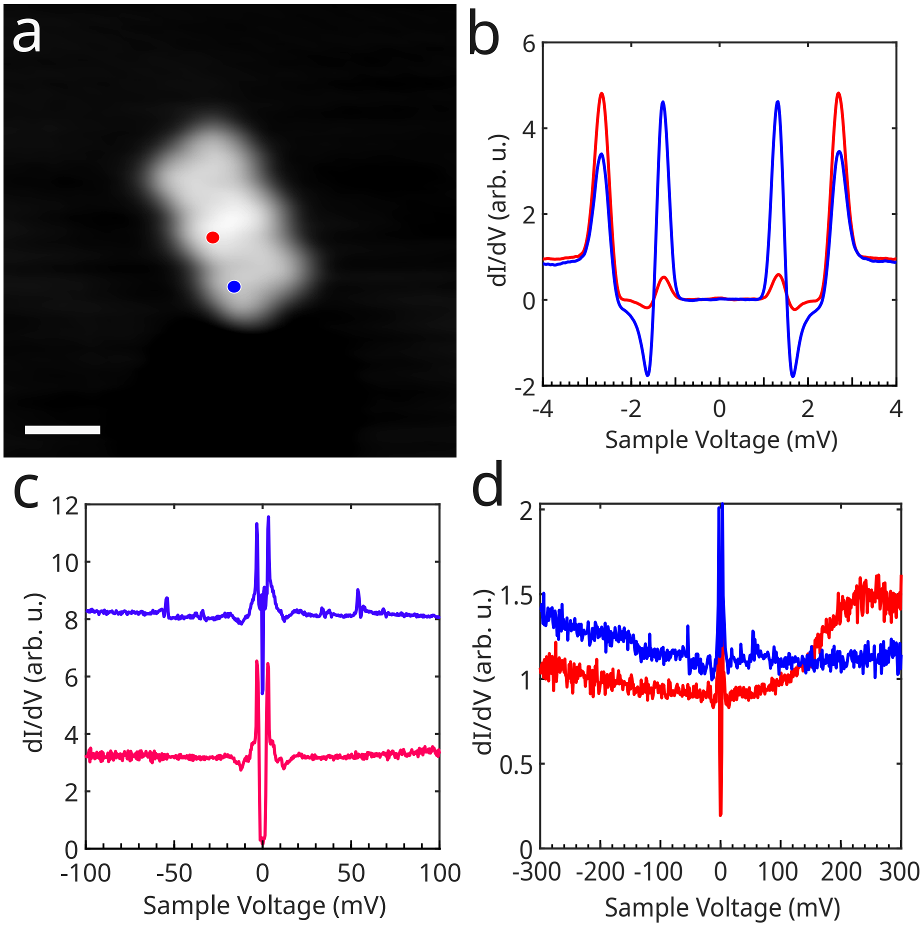

Supplementary Figure 11. (**a**) STM image of a trimer chain formed by lateral manipulation (100 mV, 100 pA). The white scale bar represents 1 nm. (**b**) Differential conductance spectra measured at the locations marked by red and blue dots in panel **a** reveal distinct electronic features: a single YSR peak pair at $V_s = \pm 1.4\,\mathrm{mV}$ for the molecule at the end (blue curve), and a faint YSR peak pair for the central molecule (red curve) that originates from the extension of YSR signals from the molecules at the end. Spectra parameters: $V_s = 5\,\mathrm{mV}$, $I_t = 0.1\,\mathrm{nA}$, $A_{mod} = 0.03\,\mathrm{mV}$ and $f = 613\,\mathrm{Hz}$. (**c**) Differential conductance spectra measured from -100 mV to 100 mV at the same locations. While no spectroscopic features are observed in the central molecule (red curve), the end molecule exhibits pronounced vibrational characteristics due to YSR resonances, with distinctly high conductance peaks at ±20, ±30, and ±50 meV (blue curve). The spectra were shifted vertically for clarity. Spectra parameters: $V_s = 100\,\mathrm{mV}$, $I_t = 0.4\,\mathrm{nA}$, $A_{mod} = 1\,\mathrm{mV}$ and $f = 613\,\mathrm{Hz}$. (**d**) Differential conductance spectra measured from -300 mV to 300 mV at the same locations. The resonance at 260 mV in the central molecule (red curve) is likely attributable to the lowest unoccupied molecular orbital (LUMO) of the neutral molecule. Spectra parameters: $V_s = 300\,\mathrm{mV}$, $I_t = 0.4\,\mathrm{nA}$, $A_{mod} = 3\,\mathrm{mV}$ and $f = 613\,\mathrm{Hz}$.

* chao.li@unibas.ch
† martin.zonda@matfyz.mff.cuni.cz
‡ shi-xia.liu@unibe.ch
§ remy.pawlak@unibas.ch
¶ ernst.meyer@unibas.ch

[1] R. C. Dynes, V. Narayanamurti, and J. P. Garno, Direct measurement of quasiparticle-lifetime broadening in a strong-coupled superconductor, Phys. Rev. Lett. **41**, 1509 (1978).

[2] J.-D. Pillet, C. H. L. Quay, P. Morfin, C. Bena, A. L. Yeyati, and P. Joyez, Andreev bound states in supercurrent-carrying carbon nanotubes revealed, Nat. Phys. **6**, 965 (2010).

[3] K. J. Franke, G. Schulze, and J. I. Pascual, Competition of superconducting phenomena and Kondo screening at the nanoscale, Science **332**, 940 (2011).

[4] M. Jarrell and J. Gubernatis, Bayesian inference and the analytic continuation of imaginary-time quantum Monte Carlo data, Phys. Rep. **269**, 133 (1996).

[5] J. Kaufmann and K. Held, ana_cont: Python package for analytic continuation, Comput. Phys. Commun. **282**, 108519 (2023).

[6] D. Bergeron and A.-M. S. Tremblay, Algorithms for optimized maximum entropy and diagnostic tools for analytic continuation, Phys. Rev. E **94**, 023303 (2016).

[7] V. Meden, The Anderson–Josephson quantum dot-a theory perspective, J. Phys.: Condens. Matter **31**, 163001 (2019).

[8] L. Farinacci, G. Ahmadi, G. Reecht, M. Ruby, N. Bogdanoff, O. Peters, B. W. Heinrich, F. von Oppen, and K. J. Franke, Tuning the coupling of an individual magnetic impurity to a superconductor: Quantum phase transition and transport, Phys. Rev. Lett. **121**, 196803 (2018).

[9] M. Žonda, O. Stetsovych, R. Korytár, M. Ternes, R. Temirov, A. Raccanelli, F. S. Tautz, P. Jelínek, T. Novotný, and M. Švec, Resolving ambiguity of the Kondo temperature determination in mechanically tunable single-molecule Kondo systems, J. Phys. Chem. Lett. **12**, 6320 (2021).

[10] F. Eickhoff, B. Lechtenberg, and F. B. Anders, Effective low-energy description of the two-impurity Anderson model: RKKY interaction and quantum criticality, Phys. Rev. B **98**, 115103 (2018).

[11] J. Homberg, A. Weismann, T. Markussen, and R. Berndt, Resonance-enhanced vibrational spectroscopy of molecules on a superconductor, Phys. Rev. Lett. **129**, 116801 (2022).

[12] C. Li, J. Homberg, A. Weismann, and R. Berndt, On-surface synthesis and spectroscopy of aluminum phthalocyanine on superconducting lead, ACS Nano **16**, 16987 (2022).
